# 3D Tune-In Toolkit: An open-source library for real-time binaural spatialisation

**DOI:** 10.1371/journal.pone.0211899

**Published:** 2019-03-11

**Authors:** María Cuevas-Rodríguez, Lorenzo Picinali, Daniel González-Toledo, Carlos Garre, Ernesto de la Rubia-Cuestas, Luis Molina-Tanco, Arcadio Reyes-Lecuona

**Affiliations:** 1 Departamento de Tecnología Electrónica, Universidad de Málaga, Málaga, Spain; 2 Dyson School of Design Engineering, Imperial College London, London, United Kingdom; University College London, UNITED KINGDOM

## Abstract

The 3D Tune-In Toolkit (3DTI Toolkit) is an open-source standard C++ library which includes a binaural spatialiser. This paper presents the technical details of this renderer, outlining its architecture and describing the processes implemented in each of its components. In order to put this description into context, the basic concepts behind binaural spatialisation are reviewed through a chronology of research milestones in the field in the last 40 years. The 3DTI Toolkit renders the anechoic signal path by convolving sound sources with Head Related Impulse Responses (HRIRs), obtained by interpolating those extracted from a set that can be loaded from any file in a standard audio format. Interaural time differences are managed separately, in order to be able to customise the rendering according the head size of the listener, and to reduce comb-filtering when interpolating between different HRIRs. In addition, geometrical and frequency-dependent corrections for simulating near-field sources are included. Reverberation is computed separately using a virtual loudspeakers Ambisonic approach and convolution with Binaural Room Impulse Responses (BRIRs). In all these processes, special care has been put in avoiding audible artefacts produced by changes in gains and audio filters due to the movements of sources and of the listener. The 3DTI Toolkit performance, as well as some other relevant metrics such as non-linear distortion, are assessed and presented, followed by a comparison between the features offered by the 3DTI Toolkit and those found in other currently available open- and closed-source binaural renderers.

## Introduction

Binaural literally means relating or involving both ears. Binaural hearing refers to the ability of the auditory system to analyse the sound at the two ears, integrate the information embedded in the acoustic stimuli, and perceive sound as coming from a three-dimensional space. The various mechanisms involved in sound localisation have been widely investigated in the past [1]. Our understanding of these provides the basis for the development of binaural spatialisation tools, which allow the creation of three-dimensional soundscapes using a simple pair of headphones as a playback device.

This paper reports on the 3D Tune-In Toolkit (3DTI Toolkit), an open-source C++ library which integrates binaural spatialisation functionalities together with other audio-related features. The 3DTI Toolkit has been developed within the 3D Tune-In (http://www.3d-tune-in.eu/) EU-funded project [2] [3], which aimed at using 3D sound, visuals and gamification techniques to support people using hearing aid devices. Within the project, several applications were developed using the 3DTI Toolkit, deployed on multiple platforms (e.g. mobile, desktop or browser), tailored to different target audiences (e.g. older users or children) and scenarios (e.g. music listening or noisy environment simulation).

The reason behind the need to develop a custom, open-source, multi-platform C++ library can be found in the challenging set of requirements on real-time performance and portability, as well as on the transparency of the audio processing chain. As described in the following sections, the 3DTI Toolkit integrates in one single open-source package several techniques developed and evaluated in the last 20 years of spatial audio research. During the development stage, particular attention has been put on the time-related aspects of the spatialisation, resulting in realistic and smooth simulation of moving sound sources, both in terms of angle and distance changes.

The following section offers a technical overview of background research in binaural hearing and spatialisation, followed by a report on the rationale behind developing a custom library, which was preferred to using existing solutions. The architecture of the 3DTI Toolkit components is subsequently described, focusing on the technical innovations integrated in the processing chain. Finally, we report on the evaluation of the various features of the 3DTI Toolkit, which are compared with the ones of other available binaural spatialisation tools.

## Technical background

The human auditory sense is able to localise sound sources in the surrounding environment thanks to several localisation cues embedded in the sound arriving at the two ears. The first two are known as interaural differences (i.e. differences between the two ears), namely the Interaural Level Differences (ILDs—differences in amplitude between the signals at the two ears) and Interaural Time Differences (ITDs—differences in time of arrival between the signals at the two ears). ILDs are mainly caused by the shadowing effect of the head, and vary according to the wavelength of the acoustic stimulus. They are therefore referred to as a frequency-dependent cue, notably larger for higher frequencies if compared with low frequencies. ILDs increase for higher frequencies when the sound source is closer to the head (near-field), as described in the following Sections. ITDs are caused by the difference in distance between the source and each of the two ears. Recent studies demonstrated a frequency-dependency of ITDs, which is though still considered non-relevant from a perceptual point of view [4].

In addition to the interaural differences, humans also make use of monaural cues (i.e. not related with differences between the two ears). The outer ear, i.e. the structures of the pinna and the external ear canal, together with other elements such as the shoulders and torso, form a set of direction-dependent filters, which vary according to the angle of incidence of the sound. Sources at different locations activate different direction-dependent filtering, resulting in modifications of the spectrum of the signals at the two ears. These are then interpreted by the hearing system in order to establish the location of the various sound sources.

### HRTF and HRIR

Both interaural and monaural cues are embedded in what is known as a Head-Related Transfer Function (HRTF—if expressed in the frequency domain) or Head-Related Impulse Response (HRIR—if expressed in the time domain), which characterizes how the ears receive a sound from a given point in space, generally specified in terms of azimuth (angle on the horizontal plane, positive going anti-clockwise), elevation (angle on the vertical/median plane, positive going upwards) and distance. A set of HRTF measurements or estimations at various locations around the listener’s head (possibly uniformly spaced at given distances), represents a full characterisation of the cues used by a specific human listener for localising a sound source in the surrounding environment. For simplicity, in this paper we use the term HRTF for the full transfer function and, by extension, the full set of measurements. We then use the term HRIR to refer to each of the individual measurements or estimations of this function at various locations, which together characterise the HRTF.

### Binaural spatialisation

The theories at the basis of the binaural spatialisation technique are not particularly recent [5]. The first binaural recording dates back to the end of the 19^*th*^ Century, the Théâtrophone [6]. However, it is only within the last twenty years that the increase in the calculation power of personal computers enabled an accurate real-time simulation of three-dimensional sound-field over headphones.

The aim of binaural simulation is to provide the listener with the impression that sound is located at a specific point in the three-dimensional space, all through a pair of standard headphones. Techniques exist for allowing binaural spatialisation to work over a pair of loudspeakers; these are usually referred-to as Transaural spatialisation [7], and are beyond the scope of this paper.

The 3D characteristics of the sound can be captured during recording with special hardware (i.e. a dummy head microphone), or simulated in post-production via spatialisation techniques (i.e. binaural spatialisation). Generating binaural signals is generally based on convolving a monaural signal with HRTFs, which, as outlined earlier, model the directional filtering of the incoming signal due to the properties of the listener’s pinna, head and body shape [8]. HRTFs are generally measured from a dummy head microphone [9], or from real people [10], in anechoic environments. The acquisition of an individualized HRTF is a very time consuming and tedious task that implies an expensive and complex setup [11]. There are several publicly available databases that offer HRTFs of a dummy head manikin and different subjects, some examples as listed below in chronological order: KEMAR (the first database of the KEMAR manikin) [9], CIPIC (45 HRTFs) [12], LISTEN (about 50 HRTFs available) [13], ARI (132 HRTFs) [14], FIU (15 HRTFs) [15], RIEC (105 HRTFs) [16] and SADIE II (20 HRTFs) [17]. The equivalent of an HRTF, but measured in reverberant environments, is known as Binaural Room Impulse Response (BRIR) [18]. Direct convolution with BRIRs is the most common technique for performing binaural spatialisation in reverberant environments, but not the only one, nor the most efficient, as described in the following sections.

This section contains a chronology of research milestones in the field of binaural spatialisation in the last 40 years. For a non-research oriented review, see [19]. For a history of binaural recordings, see [20].

In the early and late nineties, Møller and Hammershøi carried out and published a large amount of work on HRTFs in general, from the measurement methodology (i.e. where to position the microphone in the ear canal when measuring HRTFs) to the binaural playback system, looking also at audio transmission models inside and outside the ear canal [21, 22].

In the same period, Begault and Wenzel did some foundational research on the binaural technique and its applications [23], starting to look at different factors influencing spatial perception in virtual acoustic simulations using HRTFs [24]. In that study, a comparison between the impact of head tracking, reverberation and individualised HRTFs on the realism and localisation accuracy of a virtual speech source was carried out, outlining the importance of the first two when looking at sound source externalisation (i.e. the sources are localised outside the head, and not inside it as it often happens during headphones playback [25]).

Several other studies in those years investigated the possible causes for bad sound source externalisation (IHL—Inside-the-Head Locatedness). Bronkhorst and Houtgast [26] looked at the effects of room acoustic simulation on IHL. Hartmann and Wittmberg [27] extended the research to headphones calibration, followed by Kim and Choi [28]. Inanaga and colleagues, following the foundational work of Wallach [29], looked at the importance of dynamic localisation cues [30].

In 1997 Jens Blauert published “*Spatial hearing: the psychophysics of human sound localization*” [1], which is considered by many the most important textbook in the area of spatial hearing perception. Blauert’s book reviews the most relevant studies carried out in the area of spatial hearing and sound sources localisation, and set the grounds for new challenges in spatial hearing research and auditory Virtual Reality (VR).

HRTFs are normally measured at a fixed distance from the subject or dummy head microphone (usually within the range of 1 to 2 metres). The measurement of HRTFs at different distances is rather impractical and time-consuming. Various studies coordinated by Brungart investigated the changes within HRTFs for near-field sound sources [31–33], followed by studies from Lentz and colleagues looking at near-field HRTF synthesis [34], and from Romblom and Cook on compensating far-field HRTFs for near-field simulations [35].

Another widely investigated matter in binaural spatialisation is the interpolation of HRTFs, in order to simulate the movement of sound sources to locations where the HRTF has not been measured, and allow for head movement tracking. Various approaches have been implemented and tested, from time-domain [36] to frequency-domain interpolation [37], looking also at decompositions based on principal component analysis [38] and spherical harmonics [39].

In 1996, McKaeg and McGrath from Lake DSP investigated the problem of HRTF interpolation together with the one related with the computational complexity of real-time binaural processing [40]. Their solution was based on a virtual-Ambisonic approach. Ambisonic is a technique based on spherical harmonics functions, which allows recording, synthesis and playback of full-sphere surround sound [41]. Sound sources are encoded in an Ambisonic format (generally expressed as a given *order*, e.g. 1^*st*^ order Ambisonic, with higher orders corresponding to an increased spatial accuracy), and then decoded on a given set of loudspeakers, each one at a different location around the listener. Virtual-Ambisonic consists in spatialising the sound sources using the Ambisonic technique on a series of virtual loudspeakers, each then rendered in the binaural domain through convolution with fixed HRTFs. A similar approach was also followed by Noisternig and colleagues [42].

In 1995 IRCAM developed SPAT, the first commercially available high-quality real-time spatial sound processor, which included binaural spatialisation functionalities [43]. IRCAM released several updates since then, integrating various additional functionalities, such as HRTF selection, binaural reverberation and sound source directivity, celebrating its 20-years anniversary with a position paper on the past, present and future of binaural spatialisation [44]. 10 years after the release of SPAT, the Institut für Elektronische Musik und Akustik (IEM) released the IEM open library for binaural rendering in Pure Data [45].

An interesting area of research within binaural spatialisation has been HRTF synthesis. Despite most (if not all) of the HRTFs used nowadays within binaural spatialisation tools are measured ones, several research studies in the past 20 years have attempted to efficiently and effectively synthesize HRTFs. Seminal work was done by Duda and Algazi on spherical head models [46, 47], on snowman models (i.e. adding the torso to the spherical head) [48, 49], and on adaptable ellipsoidal head models (i.e replacing the spherical head model with an ellipsoid) [50]. Katz worked at HRTF synthesis using Boundary Element Method (BEM), synthesizing HRTFs from 3D computational models [51], followed by the work of Khahana [52]. A similar approach was followed by Fels and Vorländer, which used BEM for investigating the respective contributions of head, pinna and torso on HRTF components [53].

Looking more at the perceptual side of things, studies have been done on investigating the differences between spatial hearing in real and virtual conditions. Kulkarni and Colburn attempted to create a binaural renderer so that virtual sound sources could no longer be discriminated from real ones [54]. Similarly, Romigh and Brungart [55] looked at localisation accuracy using binaural spatialisation, which was judged to be equivalent to the free-field condition when using individually-measured HRTFs.

Another very productive area of research has been HRTF adaptation. Studies from Hofman and colleagues showed that the human central auditory system can adapt to localise sound sources using a new HRTF [56] (“*the auditory system is able to modify its spatial decoding to learn the spatial mapping of another individual*” [57]). Katz and colleagues carried out several studies on the topic of HRTF selection (i.e. is it possible to select a best-fitting HRTF from a set of HRTFs measured from other individuals?) and learning [58–60], confirming that rapid adaptation to non-individualised HRTFs is possible through short-term interactions within an audio-kinaesthetic Virtual Auditory Environment (VAE) platform.

Other groups and individuals that have not been mentioned above have done extensive work on spatial hearing and binaural rendering. Pulkki and his team at Aalto University carried out research on the development of spatial audio reproduction techniques, looking in depth at spatial hearing perception in complex audio-visual environments, and creating functional models of the auditory brain mechanisms [61]. Nicol looked extensively at HRTF individualisation, decomposition and modelling, and recently published a collection of studies on the topic [57].

Additional relevant research milestones to complete this overview include the work done by the BiLi project (Binaural Listening—http://www.bili-project.org/), the creation of the SOFA HRTF exchange format [62], or the Anaglyph project, which encompasses the results of over a decade of spatial hearing research by Katz and colleagues [63].

The 3DTI Toolkit builds upon the research presented in this section, integrating techniques and procedures developed by various researchers and groups in the last 30 years in one single library. Early descriptions of the Toolkit were presented in 2017 [64] as technical report paper and 2018 [65]. These publications (one of which was a poster) included only high-level descriptions of the first prototype releases of the 3DTI Toolkit, without reporting in-depth literature review, overview and analysis of the various algorithms and components.

It is true that with the uprising of VR in the last years, a large number of binaural rendering tools has been released (a complete overview can be found in the following Sections). Nevertheless, the main motivation for the development of a custom binaural spatialisation library was the need for several features which, all together, were not found to be available in other existing tools:

- Support for multiple platforms, including web.
- Full 3D placement and movement of sources and listener, including near- and far-field simulation.
- Smooth behaviour in dynamic situations.
- Customization of HRTFs.
- spatialised reverberation simulation.

The implementation of all these functionalities within an open-source tool provides the full control on the spatialisation process, as well as it opens it up for future developments within the 3D audio communities.

## 3DTI Toolkit components and structure

**Fig 1:**
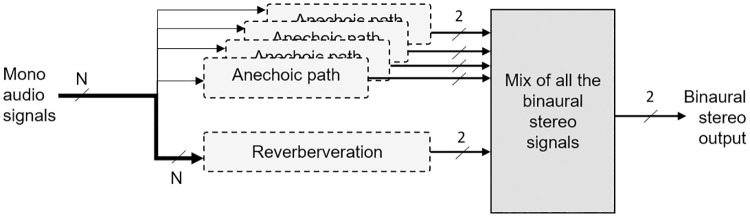
High level 3DTI Toolkit binaural process structure.

The 3DTI Toolkit approach to binaural spatialisation decouples the anechoic path simulation from the simulation of the environment (Fig 1). The simulation of the anechoic path is independently computed for every source, which allows to maintain high spatial accuracy, using direct HRTF convolution for each source. The simulation of reverberation is based on BRIR convolution. However, a virtual Ambisonic approximation has been used to reduce the computational cost, as room impulse responses may be very long. This structure allows a very high spatial resolution for the anechoic path (each source is processed in an independent anechoic path), and an efficient simulation of the reverberation, which is generated for all sources at the same time, while keeping certain location-dependent characteristics.

**Fig 2:**
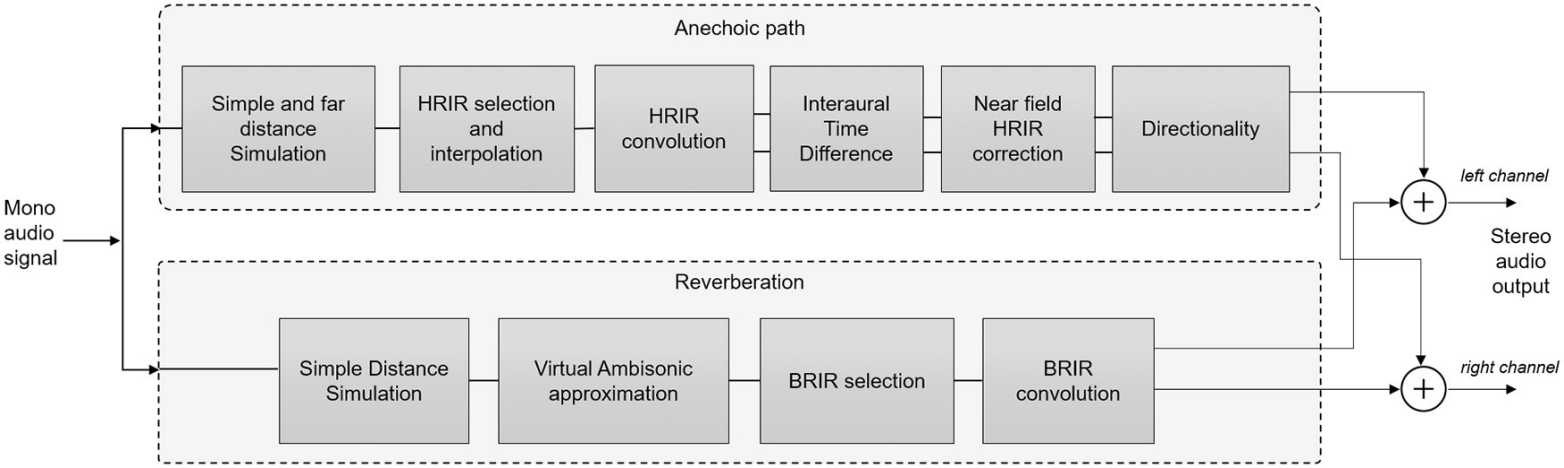
3DTI Toolkit binaural structure in detail.

Considering the challenges and requirements described above, the anechoic path and the reverberation have been designed as detailed in Fig 2, where the process chain for a single source is presented.

The anechoic path simulation is carried out through the following steps. (1) a distance-dependent attenuation is applied to the source based on the acoustic power law, which includes also a frequency-dependent far-field distance simulation for sources further than 15 meters. (2) the HRIR for the convolution is obtained by interpolating (barycentric interpolation) among a set of HRIRs selected from the full set. ITDs have been previously removed from each HRIR, in order to reduce comb filtering effects and also to allow for customisation according to the listener’s head circumference, as explained later. (3) the sound source signal is convolved with the corresponding HRIR. (4) the ITD is then computed and added to the signals, and (5) the ILD corrections for near-field simulation are applied. (6) finally, the simulation of the directionality features of hearing aids is carried out.

The reverberation simulation is also carried out in several steps which run in parallel with the anechoic path. (1) in the first instance, as for the direct path, a distance-dependent attenuation is applied to the source. This is independent from the anechoic attenuation, in order to allow, at a later stage, to modify the direct-to-reflected signal ratio in the final binaural stereo output. (2) all sources are subsequently encoded into 1^st^ Order Ambisonic B-Format signals (W, X, Y and Z channels). (3) finally, these channels are convolved with an Ambisonic-encoded version of the BRIRs, which do not contain the direct sound, only reflections.

Both anechoic path and reverberation consider 3D locations for the sources, and both the location and orientation of the listener. A set of classes to support geometric calculations have been implemented in the library for this. The Toolkit computes both polar and inter-aural spherical coordinates of each source relative to the listener. It also provides classes for handling convention-safe rigid transformations, including location and orientation. Finally, it also supports user-configurable frame sizes and sample rates. This allows to achieve very low latencies when a small frame size is selected, assuming a higher computational cost (see Real-Time performance subsection for more details).

This flexible and modular structure allows the use of each component independently, as well as simple implementation and integration of new rendering methods. Each of the components displayed in Fig 2 is described in detail in the following Section.

## 3DTI Toolkit audio rendering

Every processing module implemented in the Toolkit employs the so-called object-based paradigm, where the audio signals are stored as audio objects with associated information, such as source locations and other parameters.

As outlined before, the 3DTI Toolkit implements several rendering methods for performing full real-time binaural spatialisation. Working in real-time means not only that the sound streams have to be processed frame by frame, as they are generated, but also that the position of each source and of the listener may be different in every frame. This implies that all the filters may also have to change in every frame, potentially producing artefacts which ultimately have to be minimised in order to allow for a smooth and realistic simulation. In order to avoid increasing the computational cost and keeping the process as tidy as possible, the 3DTI Toolkit does not overlap consecutive frames using cross-fading. Hence, each block has to minimise those artefacts. Within this section, these blocks of the pipeline shown in the Fig 2 are described in detail, highlighting smoothing the mechanisms which have been implemented.

### Distance simulation

Humans perform a set of rather complex processes for estimating the distance of a sound source [66]. These involve a large set of parameters which result in the modification of the sound input into the auditory system. The 3DTI Toolkit implements a simplification of these filtering processes, considering different attenuations for the anechoic path and the reverberation, and simulating additional near- and far-field effects. HRTFs measured at a given distance are therefore modified in order to simulate sound sources located in closer or farther locations.

Another available approach, different from the one implemented in the 3DTI Toolkit, is to use databases where HRTFs have been measured at different distances from the listener [67], adding a further dimension to the HRIR interpolation process. Unfortunately such databases are still rather rare, and do not generally include different sets of HRTFs. Using this approach, users would therefore be limited to employ a handful of distance-dependent HRTF databases, rather than being allowed to import any of the many fixed-distance HRTF freely available. Furthermore, using such databases would require increased computational and disk-space resources. For these reasons, the approach presented here has been chosen. Perceptual evaluations comparing both approaches are currently being carried out in collaboration with other researchers across Europe, and will soon be published.

The Table 1 summarises which effects are computed, at what distances.

**Table 1:** Summary of the distance effects in the 3D-Tune-In Toolkit, indicating in which section the distance effect is described.

Distance to sound source	< 2 m	2 to 15 m	> 15 m
**Effects**	ILD correction (anech.) + Global attenuation; Cross-ear parallax	Global attenuation; Cross-ear parallax	Low-pass filter (anech.) + Global attenuation; Cross-ear parallax
**Section**	Near-field HRIR correction	Distance simulation (Eq 1); HRIR selection and interpolation	Distance simulation (Figs 3 and 4)

A general rule is applied to compute a global attenuation of the source depending on the distance between the source and the listener *A*(*d*). A configurable attenuation *A*_*ref*_ is applied to the signal every double distance. Assuming a 0 dB attenuation at the reference distance (*A*(*d*_*ref*_) = 1), being *d*_*ref*_ the distance at which the HRTF is measured (this can be extracted from the HRTF file):

$$\begin{array}{c} A\left(d\right)=A_{ref}^{\log_{2}\left(\frac{d}{d_{ref}}\right)} \end{array}$$ (1)

When the distance between source and listener varies, a smoothing mechanism avoids sudden changes in attenuation which could produce artefacts in the sound. An adaptive attenuation value *a*_*i*_ is applied to each sample of the audio buffer, asymptotically approaching the desired new attenuation (*A*(*d*)), using the following law:

$$\begin{array}{c} a_{i}=\left(1-\rho \right)\cdot a_{i-1}+\rho \cdot A\left(d\right) \end{array}$$ (2)

where *ρ* is calculated from *t*_*a*_ (attack time), which is the time for the adaptive attenuation to reach 99% of the change, and *f*_*s*_ is the sampling frequency:

$$\begin{array}{c} \rho =1-\exp\frac{\log0.01}{t_{a}\cdot f_{s}} \end{array}$$ (3)

This attenuation is computed separately (but using the same process) for the anechoic path and the reverberation. If different *A*_*ref*_ values are chosen for the two components, the direct-to-reflected sound ratio changes according to the distance of the sound source, emulating what happens in real-life conditions. The default *A*_*ref*_ values are -6 dB for the anechoic path, and -3 dB for the reverberation, in line with the work of John Chowning when he was working on the simulation of moving sound sources in reverberant spaces [68].

**Fig 3:**
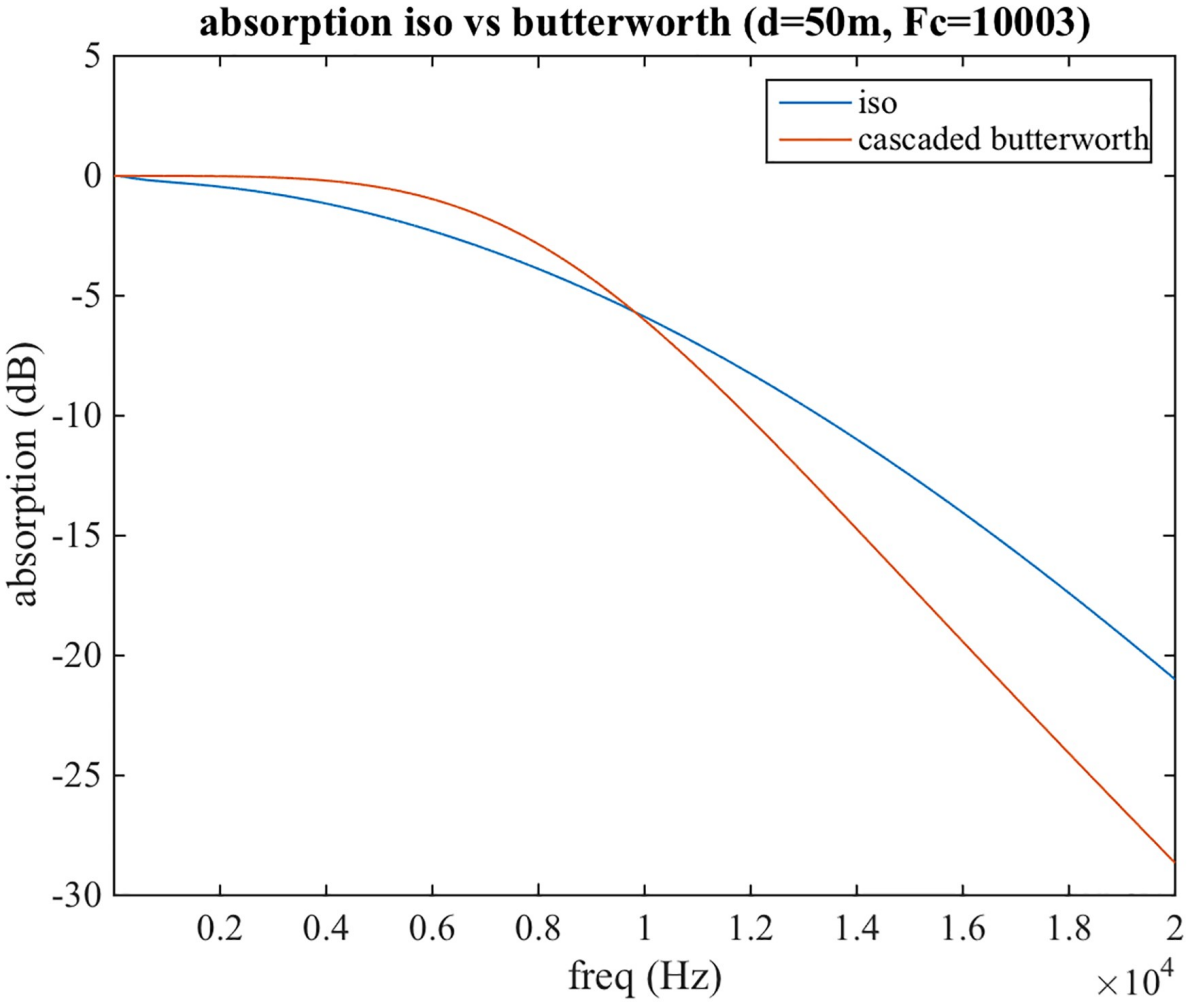
Air absorption as a function of frequency for 50 metres distance obtained from ISO9613-1 and our proposed two cascaded second order Butterworth filter.

**Fig 4:**
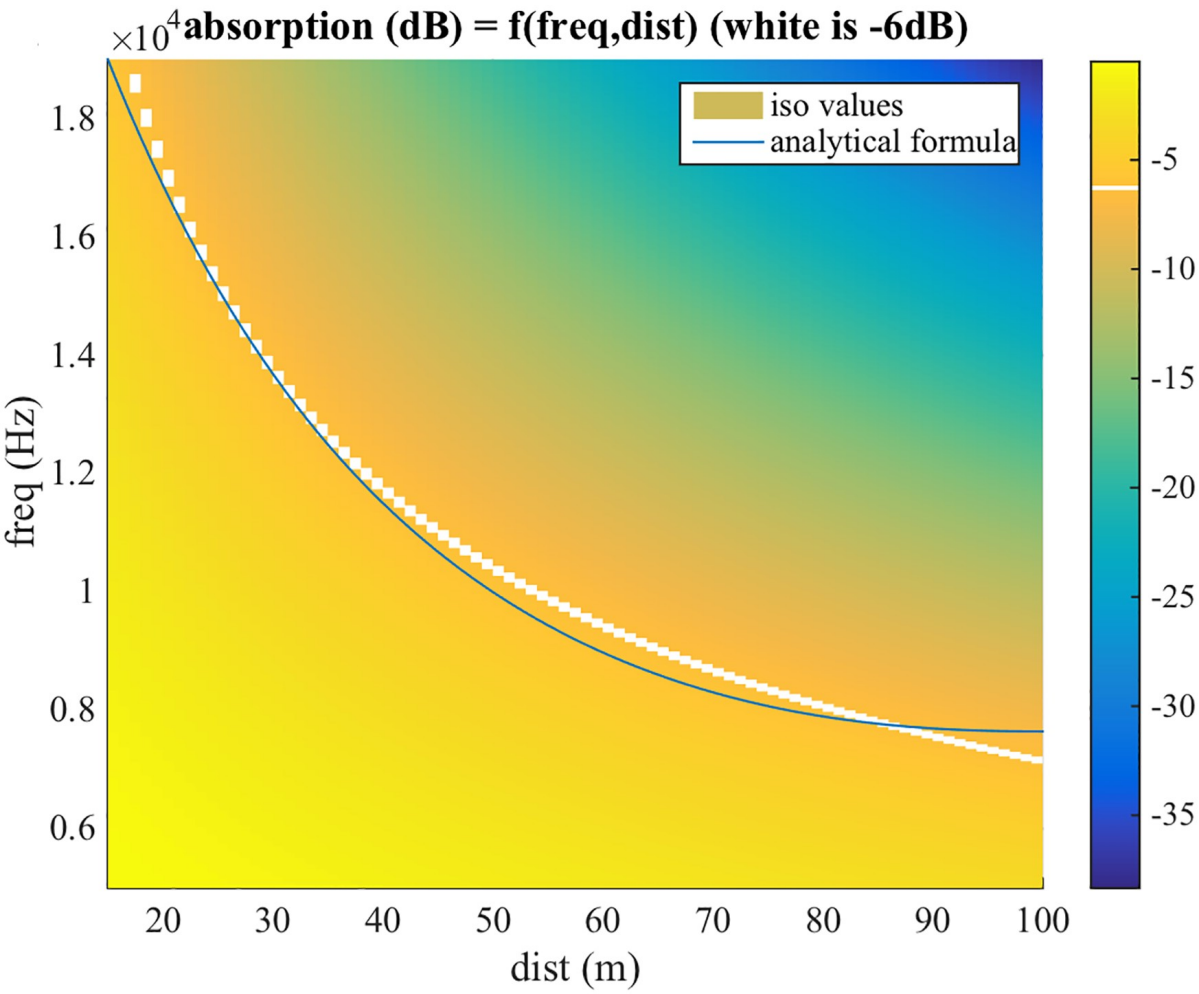
6 dB cut-off frequency from ISO9613-1 and our proposed model.

In addition to the attenuations, the following three processes are implemented only for the anechoic path. For short distances (below 2 metres), a simulation of near-field effects is added at the end of the pipeline, as a correction of the HRTF function, the details of which are described later on. For distances larger than 15m air absorption is modelled using a low pass filter designed to match data presented in the ISO9613-1 standard [69]. More specifically, two cascaded second order Butterworth low pass filters are used. These result in a roll-off of 24 dB/octave, which is a good approximation to the data reported in the ISO standard (see Fig 3). A cut-off frequency of 20 kHz has been selected as a reference for a distance of 15 meters, and is exponentially decreased as distance increases, again to match data presented in ISO9613-1 (Fig 4). For all distances, a cross-ear parallax correction is also performed, as it will be described in the following Sections.

### Convolution with HRIR and BRIR

Anechoic spatialisation within the 3DTI Toolkit is performed by convolving the sound signals with the HRIR corresponding to the desired location. Similarly, for the reverberation, a number of convolutions are performed with BRIRs.

In both cases, the 3DTI Toolkit employs a Uniformly Partition Overlap-Save (UPOLS) convolution in the frequency domain [70]. This FFT-based algorithm partitions the filter’s impulse response into a set of blocks with the same size as the input buffer signal length (N). It allows for convolution operations to be performed in a very efficient manner, especially in the case of long impulse responses (e.g. BRIRs), and more in general when using frame sizes that are shorter than the size of the impulse response.

**Fig 5:**
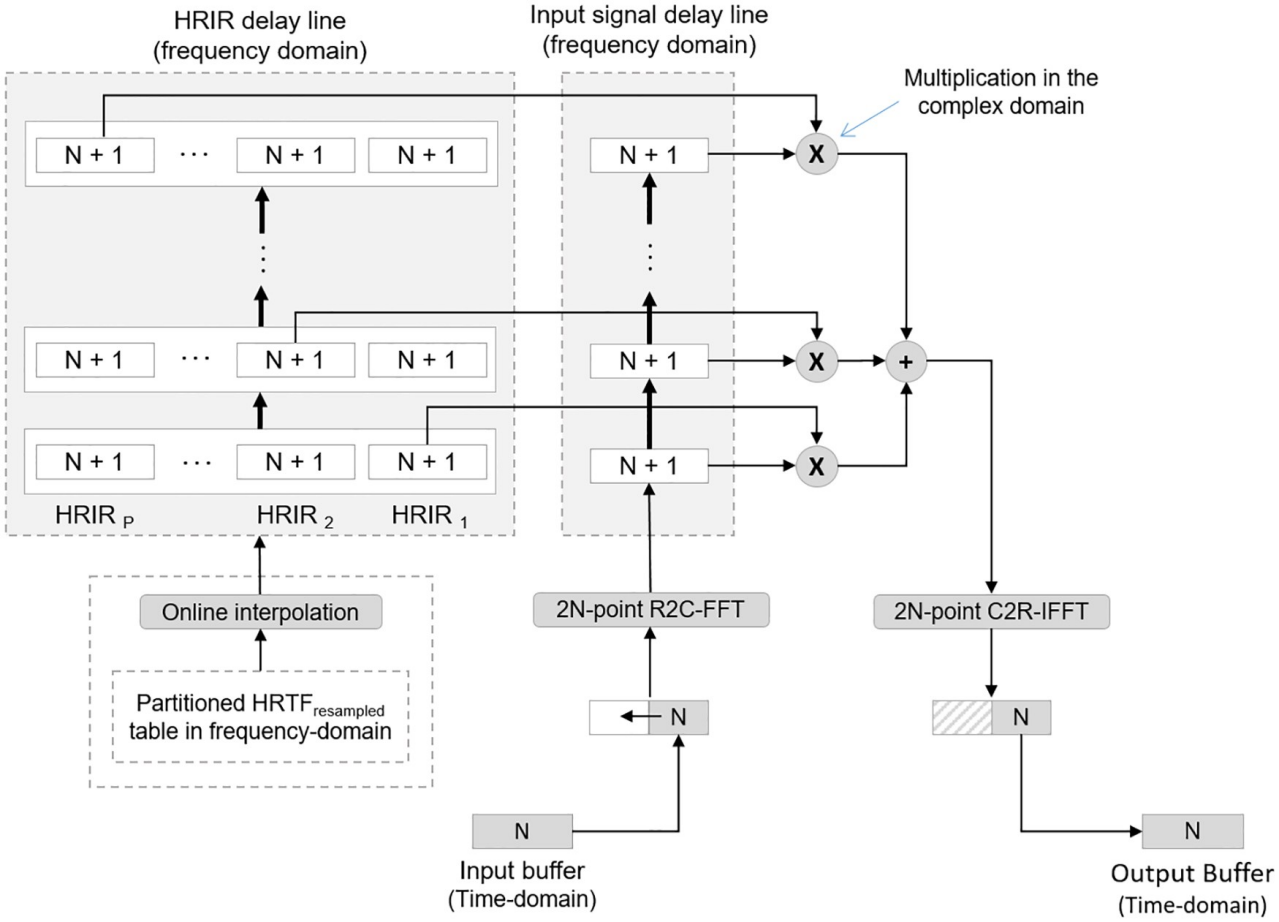
Modification of the (UPOLS) convolution algorithm of F. Wefers [70] for moving sources.

UPOLS assumes a stable impulse response, which would correspond to static sources and listener. In order to allow for the HRIR changes resulting from the movement of sources or listener, we have modified the original algorithm (see Fig 5).

Our contribution consists in adding a new delay line for the partitioned impulse response (top-left grey box in Fig 5). For every audio frame, both delays lines (HRIR and input buffer signal) are shifted up by one frame slot. Therefore, at every frame, a new HRIR, corresponding to the location of the source, is introduced in the delay line, and its first segment is multiplied with the current audio frame. In subsequent frames, that HRIR is kept, convolving its remaining segments with the audio frame as it progresses through the input delay line. As a result, the number and power of artefacts due the movement of the sources can be significantly reduced. This improvement is only relevant for convolutions with HRIRs. In the case of BRIRs, as explained later, there are no changes in the filters from frame to frame, even in the case of sources and/or listener movements.

### Anechoic path

#### HRIR selection and interpolation

The 3DTI Toolkit allows the use of any HRTF saved in the SOFA format [71] (see Section on Additional tools). We assume that ITD has been removed from HRIRs and stored in the Delay field of the SOFA file. Any matter related with the compensation of the effects potentially introduced by the equipment used for the HRTF measurement should be addressed by the user as well. HRTFs are commonly measured at a single distance from the listener, for a limited set of azimuths and elevations. In this section we describe how the 3DTI Toolkit can spatialise sources located at arbitrary coordinates, which may or may not be included in the set of measurements of the HRTF.

First of all, when the source is not located at the distance where the HRTF was measured, the acoustic parallax effect results in a modification of the relative angles between the sound source and each of the two ears, if compared with the angle between the sound source and the centre of the head. This effect is more relevant for near-field sources [72]. In order to select the most appropriate HRTF for each ear, the 3DTI Toolkit implements a cross-ear parallax correction [35]. This correction is based on calculating the projection of the vector from the ear to the source on the *HRTF sphere* (i.e. the sphere on the surface of which the HRTF was measured), giving a more accurate rendering, especially for near-field and far-field sound sources.

**Fig 6:**
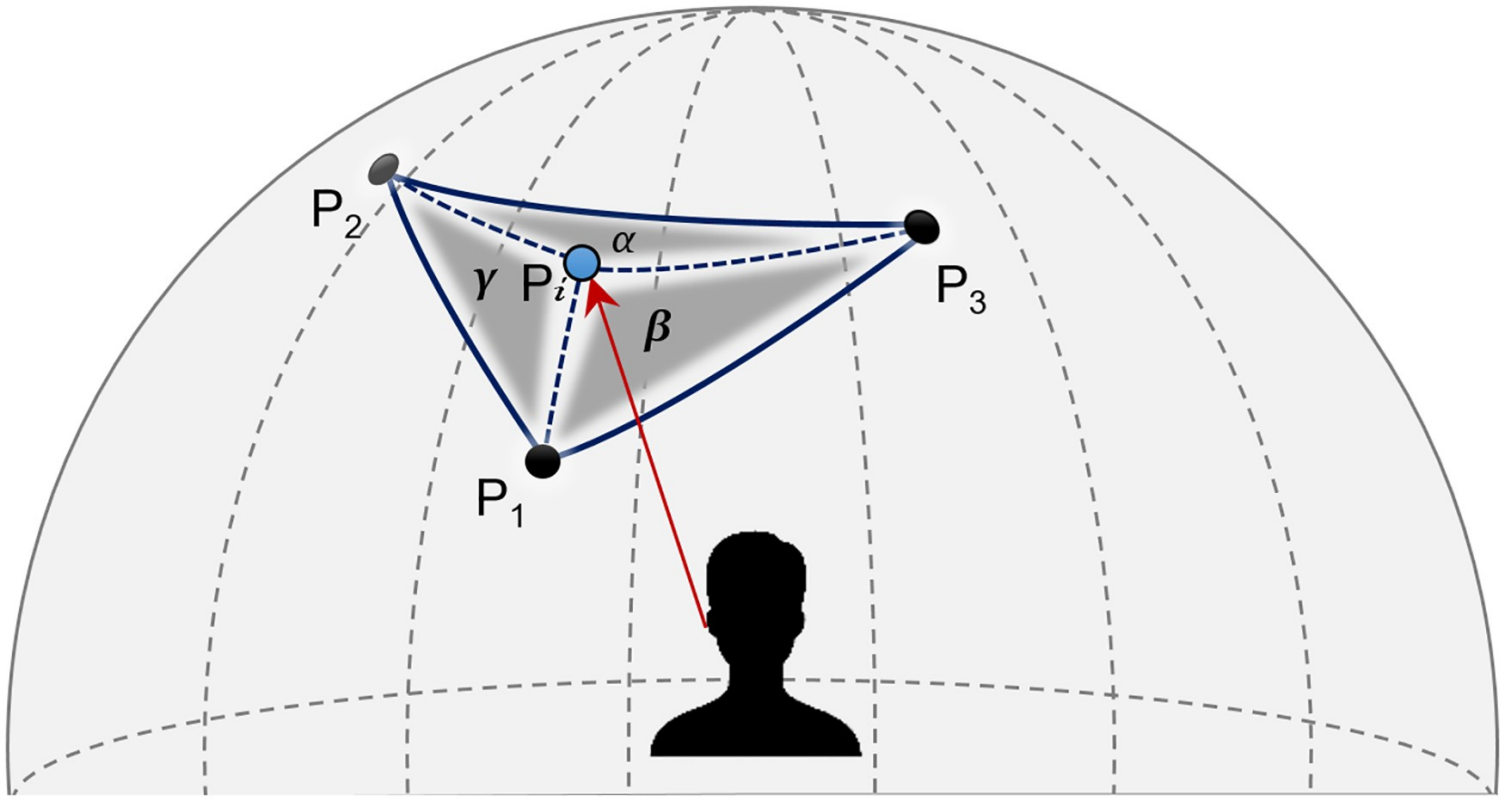
Example of barycentric interpolation in the HRTF sphere surface for the left ear.

Once obtained the projection on the HRTF sphere (*P*_*i*_) for each ear, if there is no available HRIR at that specific location, the 3DTI Toolkit selects the three nearest points at which the HRTF was measured (i.e. form a triangle containing *P*_*i*_), and performs a barycentric interpolation among the HRIRs corresponding to these three locations (see Fig 6). As mentioned in the Technical background Section, other approaches to HRIRs interpolation exist, such as the one based on decomposing HRTFs into spherical harmonics, which allows to implement sources movements and soundscape rotations directly in the spherical harmonics domain [39]. This method though requires a series of careful choices (e.g. the spherical harmonic order) in order to avoid aliasing and other phase and frequency related problems [73, 74], which could create complications when allowing users to import their own HRTFs, with custom spatial resolution and non-uniform distribution. For these reasons, we chose to employ a simpler, yet potentially less effective, approach by implementing barycentric interpolation.

In order to know the three nearest points, we need to calculate the distance *σ* between *P*_*i*_ and the rest of points included in the HRTF sphere. Considering that these are computed on the surface of a sphere, the Haversine Formula [75] (Eq 4) is used to obtain the distance between two points, where $\theta^{P_{i}}$, $\phi^{P_{i}}$, $\theta^{P_{k}}$ and $\phi^{P_{k}}$ are azimuth and elevation for the two points (*P*_*i*_ and *P*_*k*_).

$$\begin{array}{c} \sigma =2r\cdot \arccos(\sqrt[{}]{\sin^{2}(\frac{\phi^{P_{i}}-\phi^{P_{k}}}{2})+\cos\phi^{P_{i}}\cdot \cos\phi^{P_{k}}\cdot \sin^{2}(\frac{\theta^{P_{i}}-\theta^{P_{k}}}{2})}) \end{array}$$ (4)

Once the three nearest *P*_*k*_ points are known (*P*_1_, *P*_2_ and *P*_3_), the HRIR at *P*_*i*_ (${\text{HRIR}}^{P_{i}}$) is calculated interpolating among them. Some mechanisms have been already proposed for this task using bilinear interpolation among the four surrounding points [11] or a simplified version using three points [76], both assuming a regular distribution of measured positions. Gamper proposes a barycentric interpolation among the HRIRs at the vertices of a 3D tetrahedron conformed by four positions where HRIRs have been measured, which surround the point to be interpolated [77]. In a similar way, but using 2D spherical coordinates, we use a barycentric interpolation among the three positions (*P*_1_, *P*_2_ and *P*_3_) calculated above. This allows us to deal with irregular distributions of HRIRs, assuming that all measurements are at the same distance. The barycentric coefficients are calculated using Eq 5:

$$\begin{array}{c} \begin{array}{cc} \alpha & =\frac{\left(\phi^{P_{2}}-\phi^{P_{3}}\right)\cdot \left(\theta^{P_{i}}-\theta^{P_{3}}\right)+\left(\theta^{P_{3}}-\theta^{P_{2}}\right)\cdot \left(\phi^{P_{i}}-\phi^{P_{3}}\right)}{\left(\phi^{P_{2}}-\phi^{P_{3}}\right)\cdot \left(\theta^{P_{1}}-\theta^{P_{3}}\right)+\left(\theta^{P_{3}}-\theta^{P_{2}}\right)\cdot \left(\phi^{P_{1}}-\phi^{P_{3}}\right)}\\ \beta & =\frac{\left(\phi^{P_{3}}-\phi^{P_{1}}\right)\cdot \left(\theta^{P_{i}}-\theta^{P_{3}}\right)+\left(\theta^{P_{1}}-\theta^{P_{3}}\right)\cdot \left(\phi^{P_{i}}-\phi^{P_{3}}\right)}{\left(\phi^{P_{2}}-\phi^{P_{3}}\right)\cdot \left(\theta^{P_{1}}-\theta^{P_{3}}\right)+\left(\theta^{P_{3}}-\theta^{P_{2}}\right)\cdot \left(\phi^{P_{1}}-\phi^{P_{3}}\right)}\\ \gamma & =1-\alpha -\beta \end{array} \end{array}$$ (5)

where ($\theta^{P_{i}},\phi^{P_{i}}$) represent the coordinates of *P*_*i*_, previously calculated via cross-ear parallax correction, and ($\theta^{P_{1}}$, $\phi^{P_{1}}$), ($\theta^{P_{2}}$, $\phi^{P_{2}}$) and ($\theta^{P_{3}}$, $\phi^{P_{3}}$) the location of the of the nearest HRIRs (*P*_1_, *P*_2_ and *P*_3_ respectively). Finally, the ${\text{HRIR}}^{P_{i}}$ is computed, as shown in Eq 6:

$$\begin{array}{c} {\text{HRIR}}^{P_{i}}=\alpha \cdot {\text{HRIR}}^{P_{1}}+\beta \cdot {\text{HRIR}}^{P_{2}}+\gamma \cdot {\text{HRIR}}^{P_{3}} \end{array}$$ (6)

**Fig 7:**
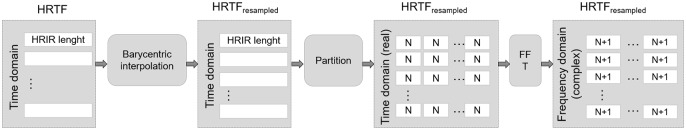
HRTF table offline process.

In practice, finding the nearest HRIR in an arbitrary set is an expensive process. For this reason, we have divided it into two separate steps. The first step (Fig 7) is performed off-line, resulting in a re-sampled HRTF table. In the first stage of this pipeline, the HRTF is re-sampled in a regular grid (5 degrees by default in both azimuth and elevation) using the barycentric interpolation method described above. At this point, HRIRs are also calculated at the poles of the sphere (elevation 90 and 270 degrees) if these are not already present in the original loaded set, interpolating among the nearest available points. Then, each HRIR is partitioned in chunks to match the input buffer length (N). This is done in order to use the modified UPOLS convolution. After this, an FFT is applied to each of the HRIR partitions and stored in memory, since the convolution is done in the frequency domain. The main purpose of this first off-line process is to get a regular HRTF table in order to simplify and accelerate the second part of the process, which is performed on-line in real-time.

The second step is performed on-line and takes the three nearest HRIRs from the re-sampled HRTF. This is now a much simpler operation, as the table has a regular basis. The barycentric interpolation is then carried out in order to get the HRIR for the specific location of the source. This interpolation is performed using partitioned HRIRs in the frequency-domain.

#### ITD simulation

**Fig 8:**
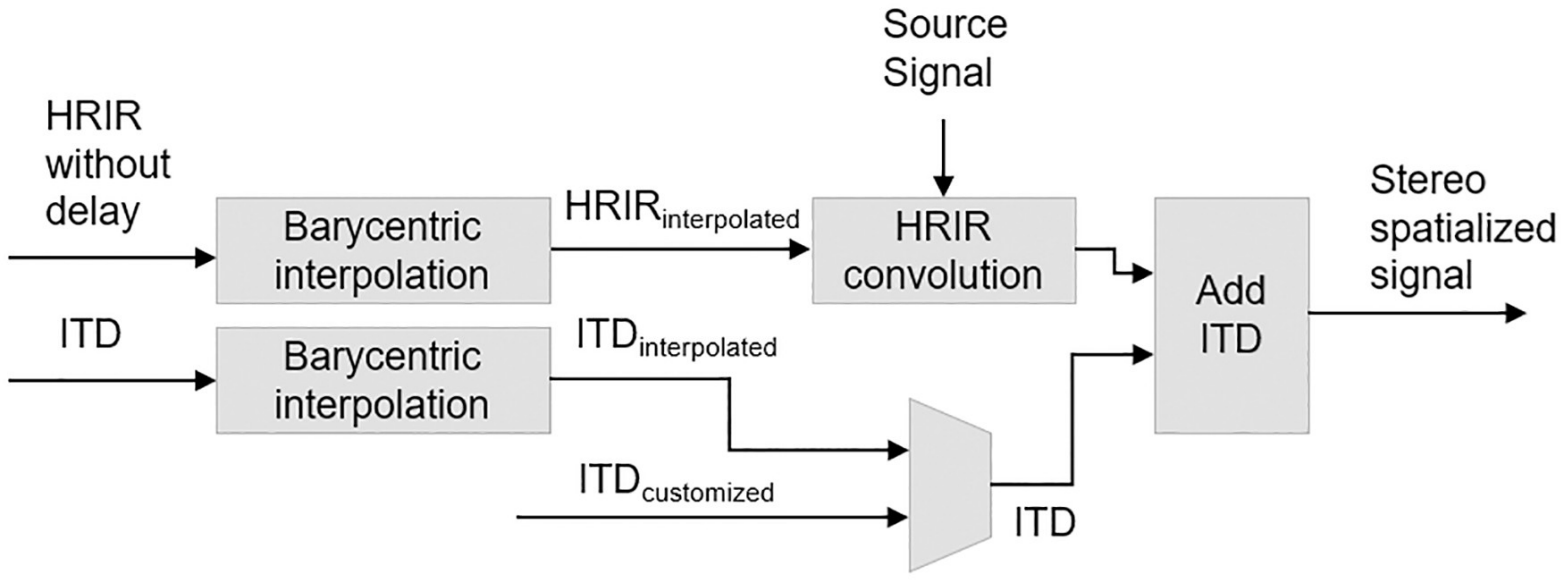
Interaural time difference simulation pipeline.

Performing interpolations between HRIRs with different ITDs can cause problems which result in audible artefacts and in a decreased rendering quality. For this reason, the 3DTI Toolkit manages the ITDs separately from the interpolation and convolution processes, using the approach shown in Fig 8.

User-imported HRIRs should be provided with ITDs stored separately, allowing to perform interpolations between synchronized HRIRs, thus reducing the comb filter effect caused by the addition of two signals with similar modules and different phases. Users can remove ITDs from their HRTFs of choice by employing different techniques [78]. A simple but effective method is to use an onset detector with a given threshold, in order to be able to identify the beginning of the HRIR, and to then remove the initial delay. This method has been employed for processing the IRCAM Listen HRTFs provided with the 3DTI Toolkit test application (see Section on Additional tools).

A more accurate description of this effect is reported in section Evaluation. The ITDs to be added after interpolation can be either estimated by interpolating among those corresponding to the three closest HRIRs, or synthesised using data about the location of the sound source (specifically the interaural azimuth) and the head circumference of the listener. In the first case, the Toolkit uses the same barycentric interpolation described previously, but this time employing the exact azimuth and elevation of the sound source (i.e. without taking into account the acoustic parallax effect). In the second case, the ITD is calculated using Eq 7, originally developed by Woodworth [79]:

$$\begin{array}{c} \text{ITD}=\frac{a}{c}\cdot \left(\theta_{I}+\sin\theta_{I}\right) \end{array}$$ (7)

where *a* is the listener’s head circumference, *c* is the speed of sound (approximately 343 m/s) and *θ*_*I*_ is the interaural azimuth (in radians, from 0 to *π*/2 for sources on listener’s left, and from *π*/2 to *π* for sources on listener’s right. It should be noticed that interaural azimuth *θ*_*I*_ is the angle between the source direction and the interaural axis, while azimuth *θ*, as previously defined, is the angle between the projection of the source direction in the horizontal plane and the front axis). The result is added as a delay only to the contra-lateral ear.

Both ITD simulation methods are available and can be freely chosen by the user.

**Fig 9:**
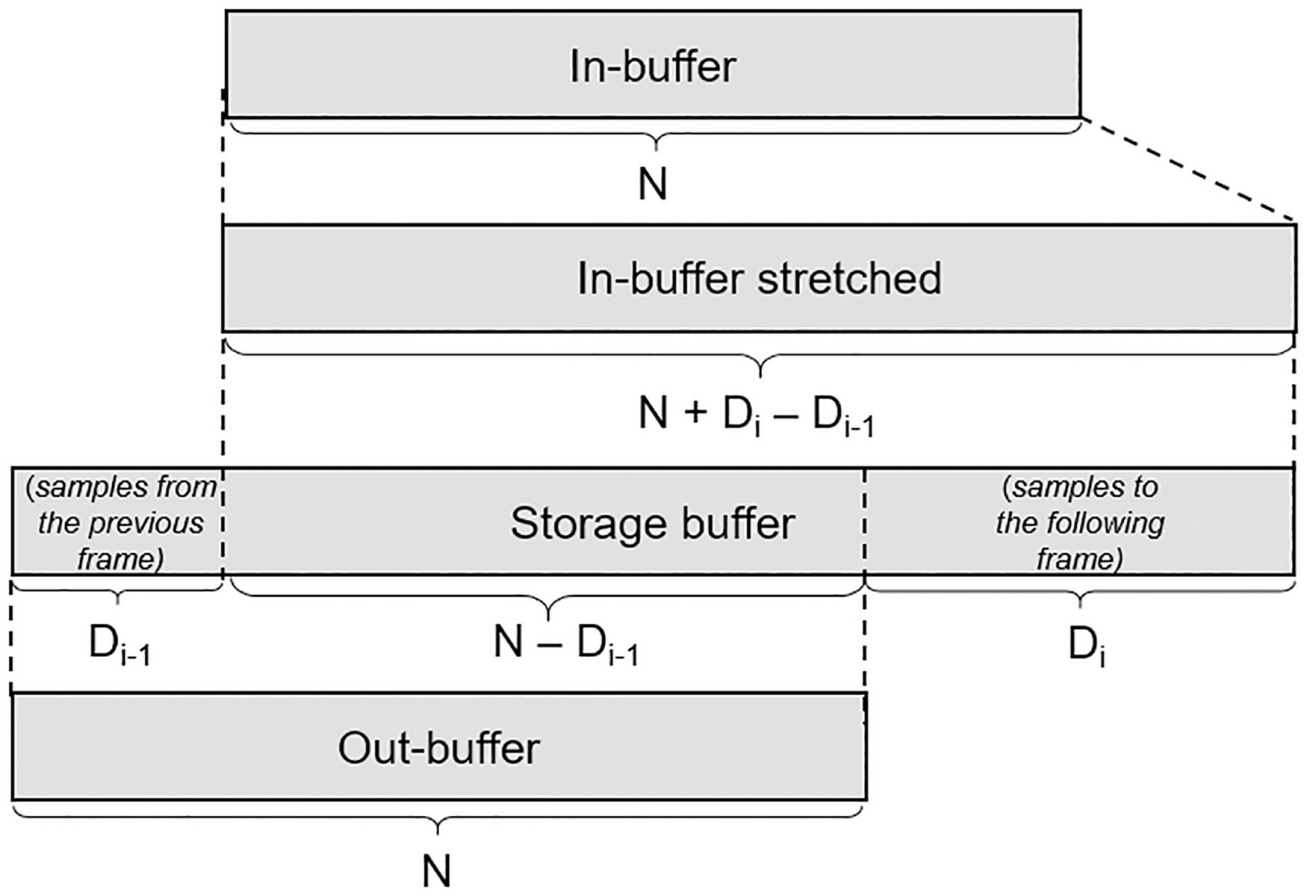
Behavior of the samples in a frame during the stretching algorithm for adding a delay.

Adding an extra delay to the contra-lateral ear presents a problem when the delay changes. This is the case when the listener’s head rotates. To avoid gaps and crackles caused by introducing a changing delay in the signal, we implemented a method to stretch or squeeze the signal. Fig 9 shows an example to illustrate the process. In this example, the current delay to be added to the frame (*D*_*i*_) is larger than the delay of the previous frame (*D*_*i*−1_), causing the input buffer to be stretched in order to fill out part of the output buffer (N-*D*_*i*−1_) and the new delay (*D*_*i*_), which will be stored to be added in the following frame. The extended buffer is re-sampled by linear interpolation among samples. The evaluation of this component is shown in section Evaluation.

#### Near-field HRIR correction

Distance perception for nearby sources is an important cue for virtual auditory simulations [80]. Most existing HRTFs databases are measured at a single distance between the listener and the source, lacking data for near-field simulations. The 3DTI Toolkit simulates sources in the near-field using models presented by Romblom in [35], which represents an extension of the conventional HRTF processing. It relies on a *difference filter* (*ILD*_difference_) that predicts the spectral differences between a near-field source and a source placed at the same azimuth and elevation angles, but at the distance where the HRTF was measured. This difference filter is based on a Spherical Head Model (SHM) presented by Duda and Martens [81], which solves the analytic problem of obtaining ILDs for a solid sphere. It is calculated as the ratio between filters given by the SHM in the far field (2 m) and the near distance which we need to simulate, as it is shown in Eq 8.

$$\begin{array}{c} {\text{ILD}}_{\text{difference}}=\frac{{\text{ILD}}_{\text{SHM}}\left(\theta_{I},d\right)}{{\text{ILD}}_{\text{SHM}}\left(\theta_{I},2m\right)} \end{array}$$ (8)

In our implementation, two biquad filters for each ear have been used, and the coefficients for these filters depend on both the distance of the sound source (*d*) and its interaural azimuth (*θ*_*I*_). These filters are pre-calculated and stored in a file as a look-up table. They can therefore be easily modified if a more complex model for near-field correction is needed. We refer to this process as an HRIR correction because it is applied in series with the HRIR selected and interpolated in the previous stages.

In both cases (HRTF convolution and high-performance mode), a problem arises when the source or the listener are moving, as the near-field correction filters have to change from frame to frame. In order to minimise this problem, and the consequent audible artefacts, at every frame each biquad filter is applied using both the previous and the new coefficients, and a linear cross-fading is performed to produce the output. This approach is not particular expensive, as these filters, which are implemented in the IIR canonical form, require only two delay cells and a minimum number of operations.

### Low-quality high-performance mode

The 3DTI Toolkit also implements a low-quality, high-performance mode, which becomes of use when, for example, computational resources are limited (e.g. on a mobile and/or web-based platform). In this case, the convolution with HRTF and all the interpolations are bypassed, and a simple ILD filter ILD_HP_(*θ*_*I*_, d) is applied. This process was designed as a 4^*th*^ order IIR filter to match the ILD of an averaged HRTF, which was estimated from seven HRTFs taken from the LISTEN database, including subject IDs 1008, 1013, 1022, 1031, 1032, 1048 and 1053. This optimized HRTF subset has been presented in [82], and was obtained and validated through a series of qualitative and quantitative tests with 45 subjects.

### Reverberation simulation

To simulate the reverberation, the 3DTI Toolkit implements the process outlined in Fig 2. The chosen approach is based on virtual Ambisonic [40] [83] and convolution with BRIR. As the anechoic path is already simulated in a parallel process line, BRIRs should not contain the direct/anechoic path here, but only the reflections, preceded by a set of zeros in order to maintain the appropriate timing between the beginning of the impulse response and the appearance of the first reflections. The number of zeros will depend on the type of environment where the BRIRs were measured/synthesized, and on the position of both the source and the listener’s microphones. A simple method for removing the direct/anechoic path from the BRIRs is to geometrically estimate the delay of the arrival of the first reflection, using this information to remove the signals that appears before that within the BRIRs, and adding an equivalent number of zeroes.

**Fig 10:**
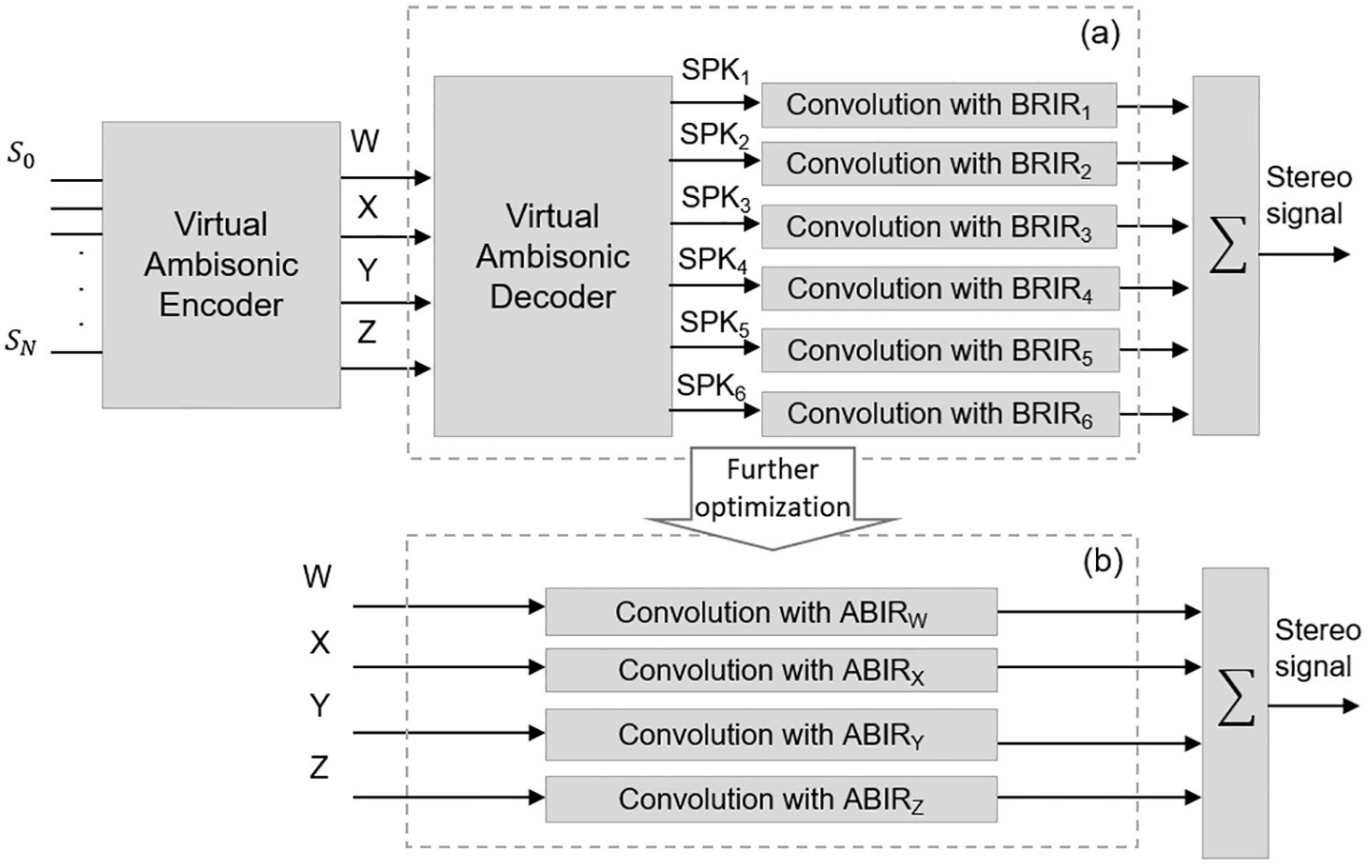
Reverberation sound simulation process. Part (a) represents the six convolutions needed for the six virtual speakers, while part (b) is a further optimization which reduces the number of needed convolutions to four.

The process is shown in more detail in Fig 10.

Using the virtual Ambisonic approach, every sound source (*S*_0_..*S*_*N*_) is encoded into a 1^st^ order Ambisonic format (B-format). Directional information of the entire soundfield is included, albeit with relatively low spatial resolution, into the first four Ambisonic channels (W, X, Y and Z), which are then decoded into a number of virtual speakers placed at a set of known locations. Finally, the virtual speakers signals are *converted* to the *reverberant binaural domain* by convolving them with the BRIRs corresponding to each of the loudspeakers locations. The virtual loudspeakers locations employed in the 3DTI Toolkit are the vertices of an octahedron: *SPK*_1_ (*θ* = 0°, *ϕ* = 0°), *SPK*_2_ (*θ* = 90°, *ϕ* = 0°), *SPK*_3_ (*θ* = 180°, *ϕ* = 0°), *SPK*_4_ (*θ* = 270°, *ϕ* = 0°), *SPK*_5_ (*ϕ* = 90°) and *SPK*_6_ (*ϕ* = 270°).

Both Ambisonic decoding and convolution are linear processes, and can be combined in order to simplify this process. The Toolkit introduces this further optimization, presented in Fig 10 in box (b). The B-Format channels (W, X, Y and Z) are directly convolved with an Ambisonic *codification* of the BRIRs for the six virtual speakers, which we refer to as Ambisonic-to-Binaural Impulse Responses (ABIRs). In this way, the number of stereo convolutions is reduced from six to four. This approach was originally introduced by [40], but in that case it was used to compute anechoic binaural spatialisation, and not BRIR-based reverberation. In [84] a similar technique was used for computing real-time binaural reverberation, but no separation between the direct and reflected signals was considered.

Using this technique, the system can handle reverberation for a virtually-unlimited number of moving sources in a full three-dimensional space, performing only four ABIR convolutions at all times. Moreover, as BRIRs and ABIRs are usually rather large, this process benefits from the use of UPOLS, as described Section Convolution with HRIR and BRIR. In order to further optimise real-time efficiency, the partition process, the encoding to ABIRs and the conversion into the frequency domain are carried out off-line, when new BRIRs are loaded.

Additionally, the 3DTI Toolkit allows to configure the number of channels used to simulate the reverberation scene, providing three different configurations in order to adapt to different needs. The first configuration is the one presented above, with four Ambisonic channels and six virtual speakers. The second configuration is included to support those situations where the BRIRs are measured only on the horizontal plane, which is a rather common case. In this case, only the W, X and Y B-format channels are computed, resulting in a reduction of the number of ABIR convolutions to three. When the source to be spatialised is located outside the horizontal plane, in order to avoid the loss of power due to the absence of virtual loudspeakers above and below the listener, the elevation, that should be encoded in the Z-channel, is computed in the X-channel using the following equations, where *θ*_*j*_ and *ϕ*_*j*_ are the azimuth and elevation of j-th source *S*_*j*_:

$$\begin{array}{c} \begin{array}{cc} W & =\sum_{j=1}^{N}S_{j}\frac{1}{\sqrt[{}]{2}}\\ X & =\sum_{j=1}^{N}S_{j}\cdot \left(\cos\theta_{j}\cdot \sin\phi_{j}+\sin\phi_{j}\right)\\ Y & =\sum_{j=1}^{N}S_{j}\cdot \sin\theta_{j}\cos\phi_{j} \end{array} \end{array}$$ (9)

The third and final configuration is included to allow efficient reverberation simulation reducing the number of convolutions to one only. In this case, the 3DTI Toolkit uses only the W B-Format channel, convolving it with a single BRIR, obtained by averaging all the available BRIRs (generally six or four).

Due to the fact that a fixed number of BRIRs are used for the reverberation simulation, and that these do not change depending on the location of the sound source, in order to simulate sources located close to boundaries within a reverberant environment (e.g. close to a wall) user-imported BRIRs need to be measured/synthesized from those locations/conditions. The 3DTI Toolkit does not offer facilities or functionalities to synthesize or modify BRIRs in order to simulate such conditions. The current implementation of the 3DTI Toolkit reverberation simulation is limited to 1^st^ order Ambisonic, but further implementations of the same method are possible increasing the Ambisonic order, and consequently the spatial resolution. We are currently working at a study to better understand the need for high spatial resolution for the simulation of reverberation in the binaural domain. Preliminary results have been published in [85].

### Directionality

The 3DTI Toolkit includes the simulation of two microphones with variable directional patterns located each on one of the listeners ears. This feature has been incorporated to allow the emulation of the directional microphones that are included, for example, in some models of hearing aids. In order to make the simulation more realistic, HRTFs recorded from hearing aids placed on a dummy head should be used in this case [86]. Moreover, as mentioned in the introduction, the Toolkit also includes modules implementing hearing loss and hearing aids simulators which can be used in combination with this feature. These are not described here, as they are out of the scope of this paper.

The implemented directionality pattern of attenuation *A*(*θ*_*F*_, *ϕ*_*F*_) is assumed to have axial symmetry around the front axis, being *θ*_*F*_ the angle to the front direction, and *ϕ*_*F*_ the rotation around that front axis. The pattern is also assumed to have no attenuation in the front direction (*A*(0, *ϕ*_*F*_) = 1). In addition, it is designed to have attenuation *δ* in the back direction as a configurable parameter. By default, a cardioid pattern is implemented:

$$\begin{array}{c} A\left(\theta_{F},\phi_{F}\right)=1-\delta +\delta \cdot \cos\left(\theta_{F}\right) \end{array}$$ (10)

This attenuation is directly applied to the anechoic path, depending on the direction of the source. To calculate the attenuation to be applied to the reverberation when directionality is enabled, the surface integral of the directionality pattern over the whole sphere *S* is calculated. This integral is applied as a summation of power, because we consider all contributions of reverberation as statistically independent:

$$\begin{array}{c} A_{reverb}=\sqrt{{∯}_{S}A^{2}\left(\theta_{F},\phi_{F}\right)dS} \end{array}$$ (11)

### Additional tools

The 3DTI Toolkit provides an optional set of libraries for allowing a simple management of the data resources (HRTFs, BRIRs and ILD models). These third party libraries are linked as Git sub-modules.

A first library allows loading HRTF and BRIR data from standard AES69-2015 (also known as SOFA) files [71]. One of the advantages of reading standard SOFA files is the wide range of existing HRTFs databases that can be loaded [87], either directly (when they provide the SOFA files), or by using the SOFA Matlab/Octave API. [88]. Our SOFA reader is based on the LibSofa library [89] for Linux/MacOS, which has been ported also to Windows.

A second library allows loading HRTFs, BRIRs and ILD filters data from custom binary formats (.3dti-hrtf,.3dti-brir and.3dti-ild, respectively). Although the binary reader is available for all platforms, it was implemented mainly to work with mobile platforms (Android, iOS) because of two reasons. Firstly, SOFA files need to be parsed, which is a slow process compared with direct serialization from the binary files to the internal data structures of the 3DTI Toolkit. Secondly, LibSofa is not ported to all platforms, and its portability is not immediate since it depends on third party libraries which need to be ported as well. The binary reader uses the C++ Cereal library for serialization [90], which is also highly portable.

**Fig 11:**
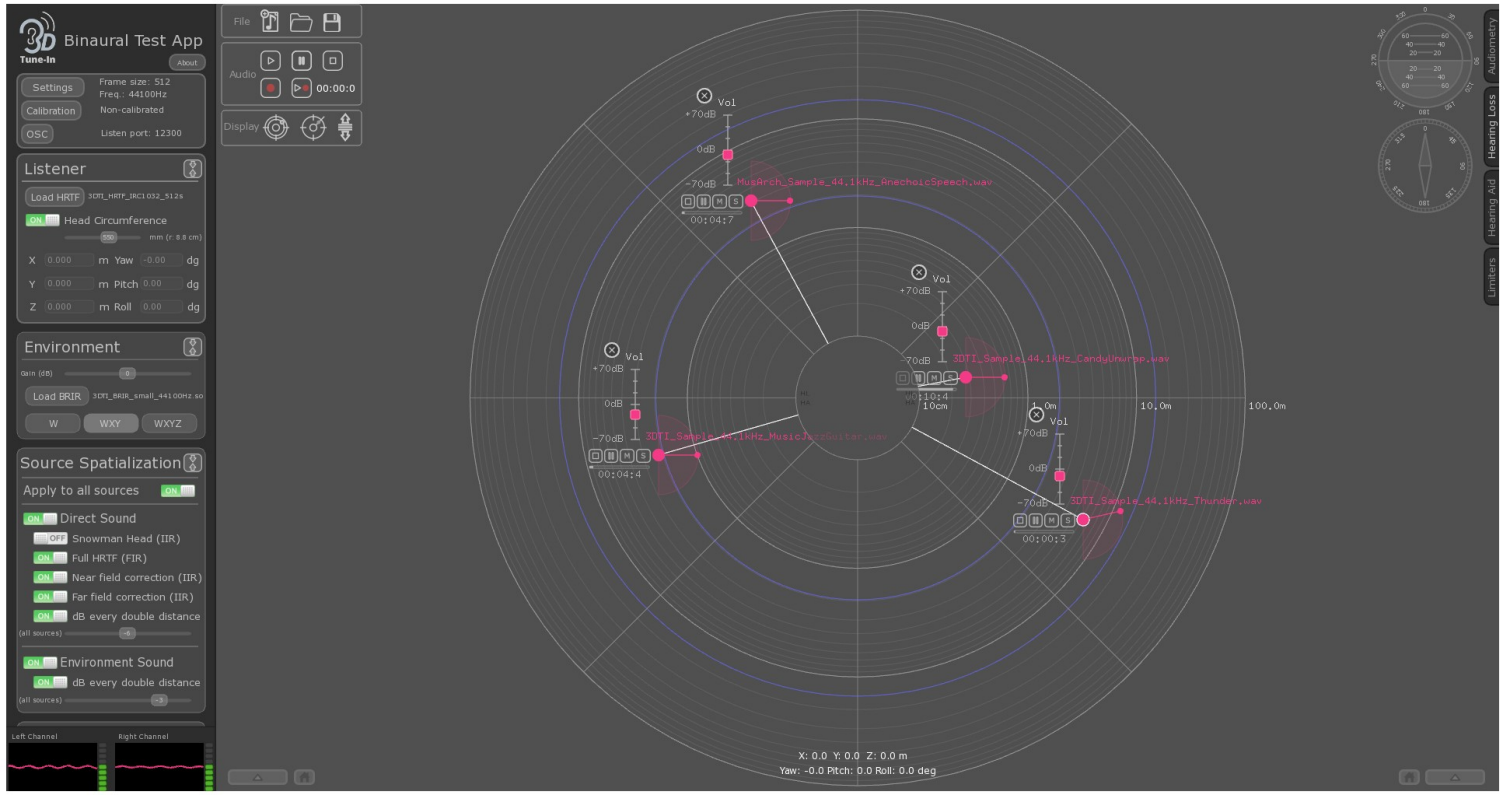
3DTI Toolkit test application snapshot.

In order to allow simple access to the various features available in the 3DTI Toolkit for testing and evaluation purposes, a demonstrator test application has been created (available for Windows, MacOS and Linux). A snapshot of the user interface can be seen in Fig 11. This test application is not open-source, but can be freely downloaded from the release page in the repository (https://github.com/3DTune-In/3dti_AudioToolkit/releases). Sources and listener positions, as well as audio playback and levels for each source independently, can also be controlled remotely via Open Sound Control (OSC—[91]). This allows the test application to be used as an audio rendered, fully and remotely controlled by other applications, such as VR visual renderers, motion tracking systems, etc.

## Evaluation

In order to evaluate how well the 3DTI Toolkit performs, we have conducted a series of tests which are presented in this Section. First, real-time performance indicators are presented to report on how many sources can be simultaneously rendered and how large can the simulated environment be (i.e. how long can the BRIR be before real-time performance is affected). Then we assess the proposed technique to interpolate HRIRs separately from the ITD, looking at spectral variations and comb filtering. Finally, measurements of non-linear distortion are presented to assess how well the 3DTI Toolkit behaves in dynamic situations.

Due to the fact that open-source tools with a set of features similar to the ones offered by the 3DTI Toolkit are not currently available, it has not yet been possible to carry out a comparative objective evaluation of the Toolkit performances. Comparisons with closed-source tools, both in terms of objective signal processing performances and subjective evaluations (e.g. spatialisation quality and realism), are being planned and will be object of further publications.

### Real-time performance

The 3D Tune-In Toolkit is designed to work on Windows, MacOS and Linux, which are not real time operating systems. This means that its process has to share CPU time with other unforeseeable processes. Therefore, its performance can be hindered by interruptions decided by the operating system to let other processes to take the CPU. For this reason, the time that the Toolkit takes to process one frame is the actual time devoted to produce the spatialised audio plus the time taken by those interruptions in case they enter during the process. If this total time exceeds the frame time, the whole audio frame will be dropped out, producing an audible artefact. All tests presented in this sections were performed on a desktop computer with Intel i7-6700 microprocessor, 3.40 GHz and RAM of 16 GB, working with Windows 10, 64 bits. A test application using the Toolkit was the only user process running in the computer besides the operating system processes. Time measurements were performed using the profiling tools included in the Toolkit, so they were integrated in the test application.

As described before, the 3DTI Toolkit processes the anechoic path and the reverberation separately. These are independent process chains and follow different approaches. While the anechoic path is rendered per source, reverberation is generated over an Ambisonic encoding of all sources, which are therefore merged together at the beginning of the process. For this reason, real-time performance is evaluated separately for both components.

**Fig 12:**
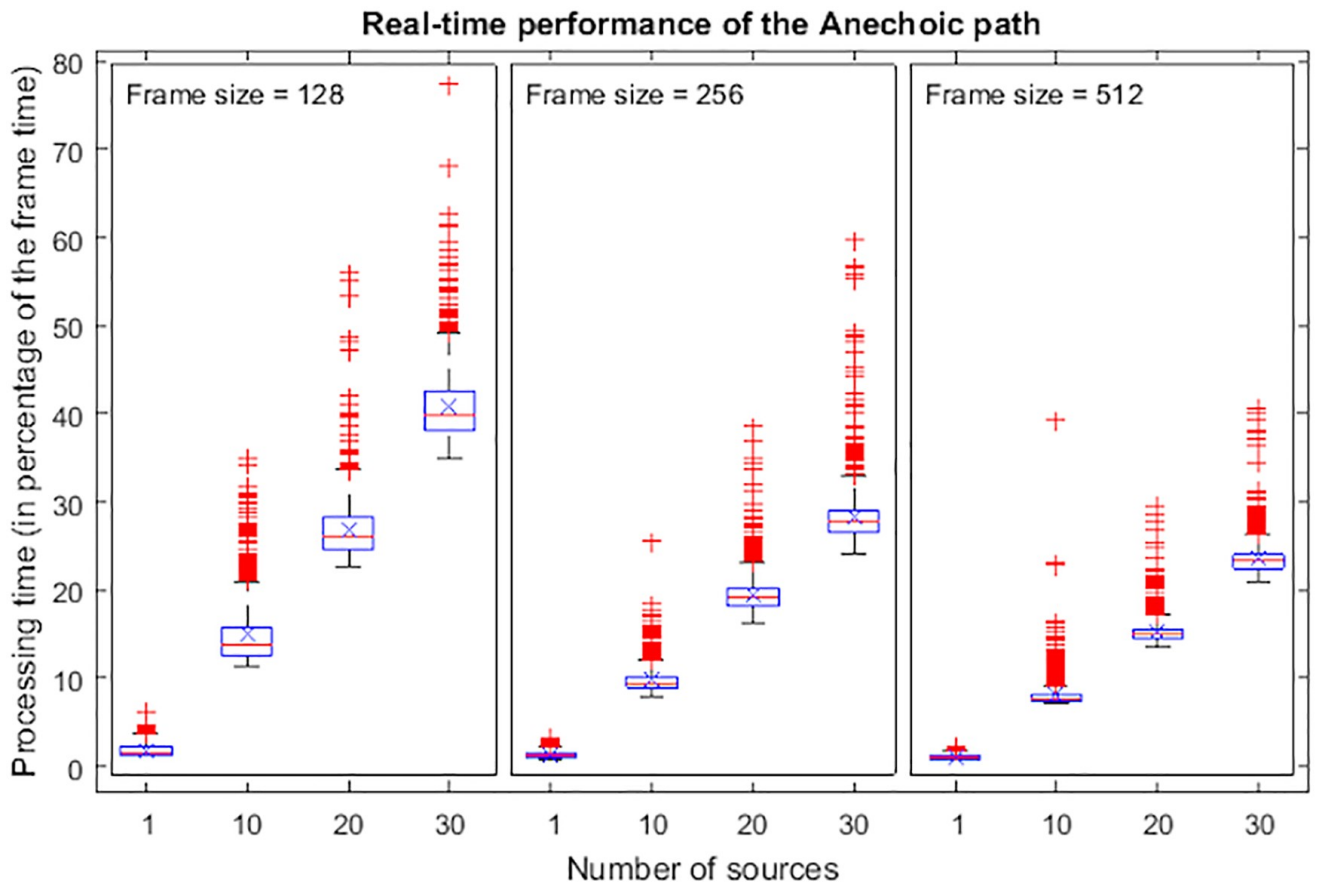
Performance of the anechoic process depending on the frame size. The horizontal axis shows number of sources and the vertical axis shows percent of total frame time.

Fig 12 shows a set of box plots of the percentage of available time in a frame taken by the Toolkit to process every frame for different number of sources and various frame sizes. This rendering was made using an HRIR of 512 samples and a sampling frequency of 44100 Hz. For each condition, 1000 frames were recorded. The boxes represents the inter-quartile range between Q1 and Q3, being the median represented with a central line. The mean has been added as a cross. As expected, this percentage, or duty cycle, mostly concentrates around their typical values, which are assumed to represent an estimation of the net processing time. It linearly increases with the number of sources, allowing to render a relatively large number of sources in an ordinary desktop computer. Moreover, the frame size has a significant influence on performance; the lower the frame size, the larger the duty cycle. There are also some outliers which we can attribute to the presence of those interruptions commented before. In these tests, no frame took longer than the available time. Hence there was no dropout.

**Fig 13:**
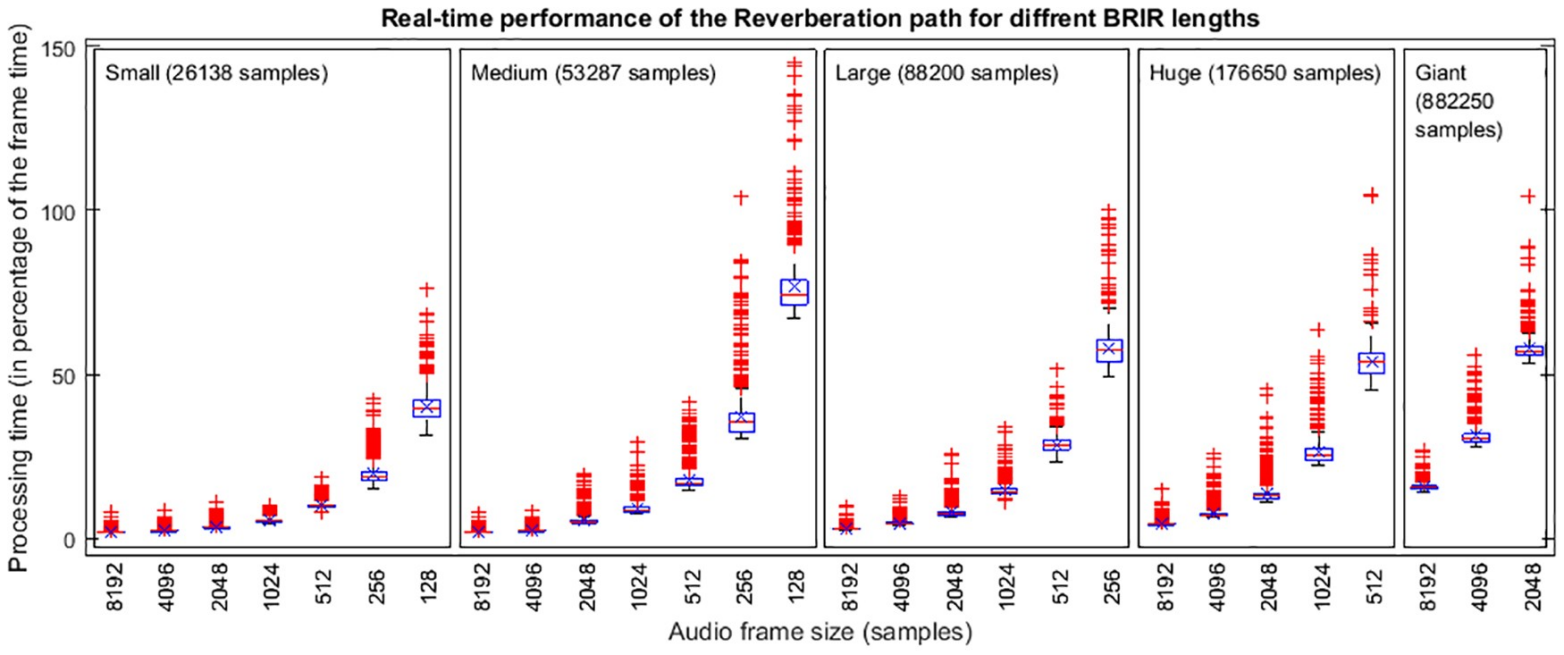
Performance of the reverberation process depending on BRIR length. The horizontal axis shows different frame sizes and the vertical axis shows percent of total frame time.

Similarly, the chart presented in Fig 13 shows the duty cycle for the reverberation process. This process is almost independent of the number of sources involved. In this test, 10 sources were used, and the duty cycle was measured for different BRIR lengths and various frame sizes. Again, 1000 frames were recorded for each condition. It can be noted that, as the frame size increases, the duty cycle decreases. For very large BRIRs and small frame sizes, it was not possible to perform the processes in real-time; these data points are therefore not reported in the chart. Moreover, for some conditions shown in Fig 13, as that for a frame size of 128 samples using the medium room, even having enough time to perform the process (around 75% of duty cycle), there are some frames which took more than 100% of the available time in the frame, producing dropouts and yielding to low quality.

A careful selection of the frame size provides the possibility of rendering with low latency very long reverberations. There is a trade-off between latency and supported computational cost. Taking a sample rate of 44100 Hz as a reference, a frame size of 128 samples implies a latency of 2.9 ms and, as it has been shown with the computer used for this test, the 3DTI Toolkit is able to render up to tens of sources with small and medium reverberation lengths (up to around 1s). On the other side, very long reverberation (we tested a very large room with a BRIR of 20 s, i.e. 882250 samples) can be rendered at the cost of sacrificing latency.

Both anechoic and reverberation processes are independent of each other, and the total computational cost can be estimated by simply summing the values for each of the two processes. As an example, referring to Figs 12 and 13, and considering a scenario with 20 sources in the large room, with a frame size of 512 samples, the anechoic path takes 15% of the CPU time on our test computer, and the reverberation path takes 29% of the CPU time. Consequently, this scenario takes 44% of the total CPU time, producing no dropouts.

### Evaluation of the HRIR interpolation technique

As described in Section HRIR selection and interpolation, the 3DTI Toolkit obtains the HRIR for the convolution process using a barycentric interpolation of the three nearest HRIRs, with the initial delay (ITD) removed. The latter operation is needed in order to minimize comb filter effects, which are produced when two signals with the same amplitude but different phases are superposed. In this section we attempt to evaluate the benefits of this process, and how the comb filtering effect is indeed avoided.

The evaluation has been done using the test application presented in Section Additional tools. A white noise source (20 minutes length) is placed at 15°, 45° and 75° of azimuth (on the left side) and 0° of elevation (relative to the listener). The HRTF number 1013 (raw version) of the IRCAM LISTEN database is used. The source is spatialised using the full HRIR from the database. Then, we spatialised again at the same locations using interpolated HRIRs from the nearest measured locations in the database, excluding the one where the source is located. This interpolation was computed in two ways: using the original database which includes ITD within the HRIR (HRTF with ITD) and the version where ITD was removed from the HRIR and included in the “Delay” field within the SOFA file (HRTF without ITD). Only the anechoic path processing is activated, disabling reverberation and distance simulation. We have recorded the system output signal during the 20 minutes, and then computed the Spectral Difference (SD) between the interpolated versions (*Y*_*HRIRinterpolated*_(*f*)) and the original one (*Y*_*HRIRoriginal*_(*f*)), as follows:

$$\begin{array}{c} SD\left(f\right)=10\cdot log_{10}\frac{|Y_{HRIRinterpolated}{\left(f\right)|}^{2}}{|Y_{HRIRoriginal}{\left(f\right)|}^{2}} \end{array}$$ (12)

**Fig 14:**
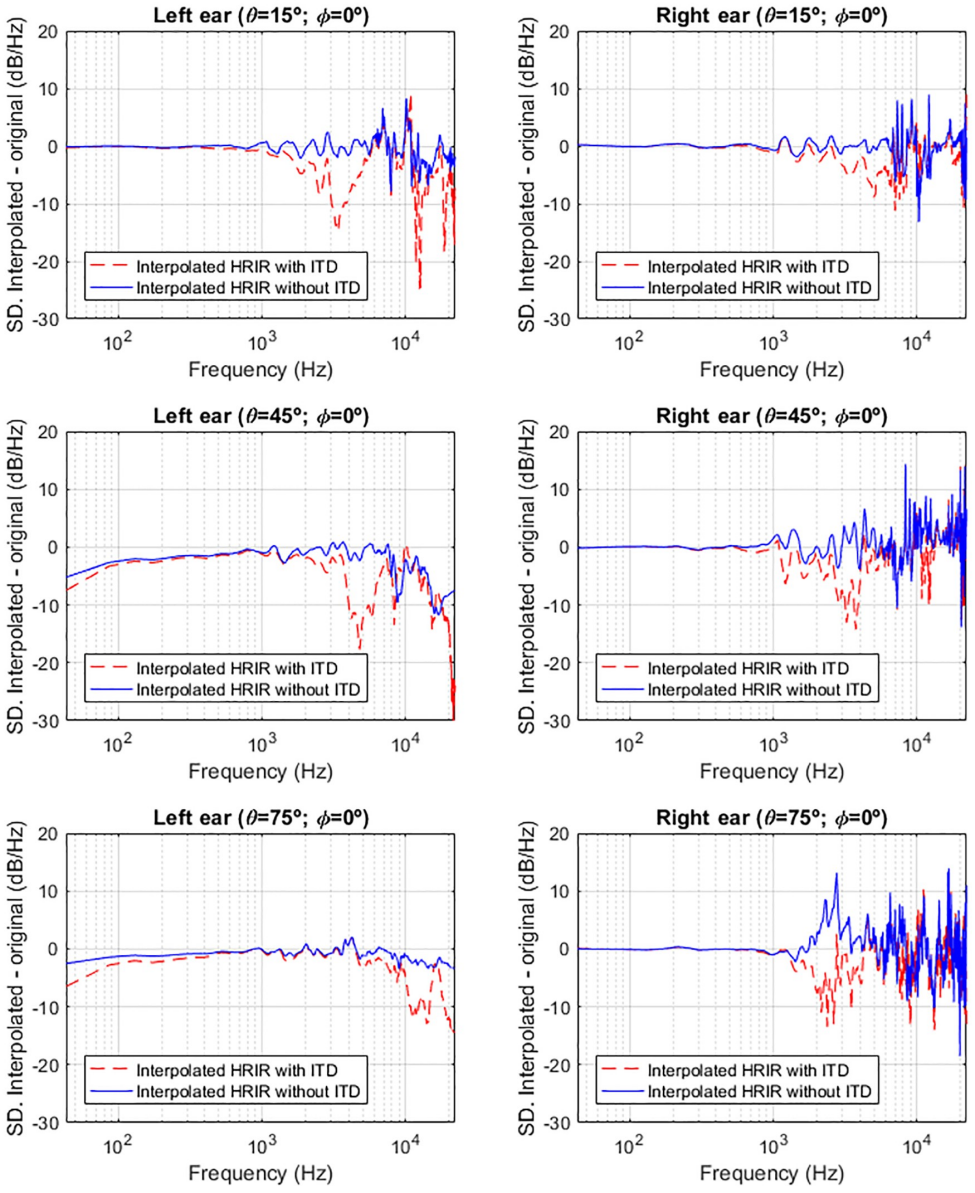
Spectral differences for white noise source at 15°, 45° and 75° of azimuth and 0° of elevation comparing two interpolation methods (with and without ITD) with the real HRIR.

Results are shown in Fig 14 for left and right channels and for the three locations. As can be seen, using the HRTF with ITD (red dashed line) produced some important colouration due to comb filtering effect, causing additional notches to appear in frequencies between 2 kHz and 6 kHz and from 10 kHz to 17 kHz. On the other hand, the use of the interpolated HRIR without ITD, computing it after the interpolation (blue line) results in lower spectral differences. We can therefore say that HRIR generated interpolating without ITD is more similar to the one of the original HRIR from the database. It is though true that there are also some spectral differences between the original HRIR and the one interpolated without ITD, but it should be considered that the comparison is being carried out between a HRIR measured in a given location, and another HRIR estimated through interpolation between HRIRs measured from two adjacent locations. A certain level of spectral discrepancies are therefore to be expected. Whether these are above or below the just-noticeable difference in terms of spectral perception, it can be verified only through perceptual evaluations which, as mentioned before, are currently being planned.

Finally, we should mention that the benefit of removing ITD from HRIRs to improve interpolations is greater for the ipsilateral ear. In the case of the contralateral ear, especially for the most lateral source (*θ* = 75°), this benefit is not as marked, but it must be taken into account that this is the HRIR which produces the lower output signal, because of ILD.

### Evaluation of the reduction of non-linear artefacts

As widely described above, the 3DTI Toolkit supports real-time 3D audio spatialisation for moving sources and listener. This implies the need to change gain, filter coefficients and delays in consecutive audio frames, resulting in discontinuities which may produce audible artefacts. Many mechanisms to avoid these problems have been implemented and described in previous sections. This section presents an evaluation of the 3DTI Toolkit dynamic behaviour, to assess how all these problems are minimised.

This analysis is based on measuring the non-linear distortion produced by the 3DTI Toolkit when a source is moving at different speeds. For this purpose, the system has been tested with a signal composed by three representative tones (859.65 Hz, 4298 Hz and 8596 Hz), estimating the percentage of the Energy out of Band (EoB), following the approach described in [92]. The frame size used in the test was 512 samples, and the sample rate 44.1 kHz. We used HRTF number 1013 from the LISTEN database (raw version). The source was moved on the horizontal plane around the listener at different distances and with different speeds for 360 frames (4.18 s). Hence, we computed the FFT of an output signal of *N* = 360 ⋅ 512 samples. The result was *Y*[*i*], composed of *N* samples as well. Then we defined the energy of frequency *f*, as the energy contained in *M* = 361 samples around *f*:

$$\begin{array}{c} E\left(f\right)=\sum_{i=i_{f}-\left(M-1\right)/2}^{i=i_{f}+\left(M-1\right)/2}{\left|Y[i]\right|}^{2}, \end{array}$$ (13)

being *i*_*f*_, the index of the sample that correspond to frequency *f*, that’s to say, *f* = *i*_*f*_ ⋅ *f*_*s*_/(2 ⋅ *N*), where *f*_*s*_ is the sampling frequency. We also define the total energy through

$$\begin{array}{c} E_{tot}=\sum_{i=0}^{i=N-1}{\left|Y[i]\right|}^{2}, \end{array}$$ (14)

Then, for the case of our three tones, the EoB, in percentage, is computed as:

$$\begin{array}{c} EoB\left(\%\right)=100\cdot \frac{E_{tot}-E\left(859.65\right)-E\left(4298\right)-E\left(8596\right)}{E_{tot}} \end{array}$$ (15)

**Fig 15:**
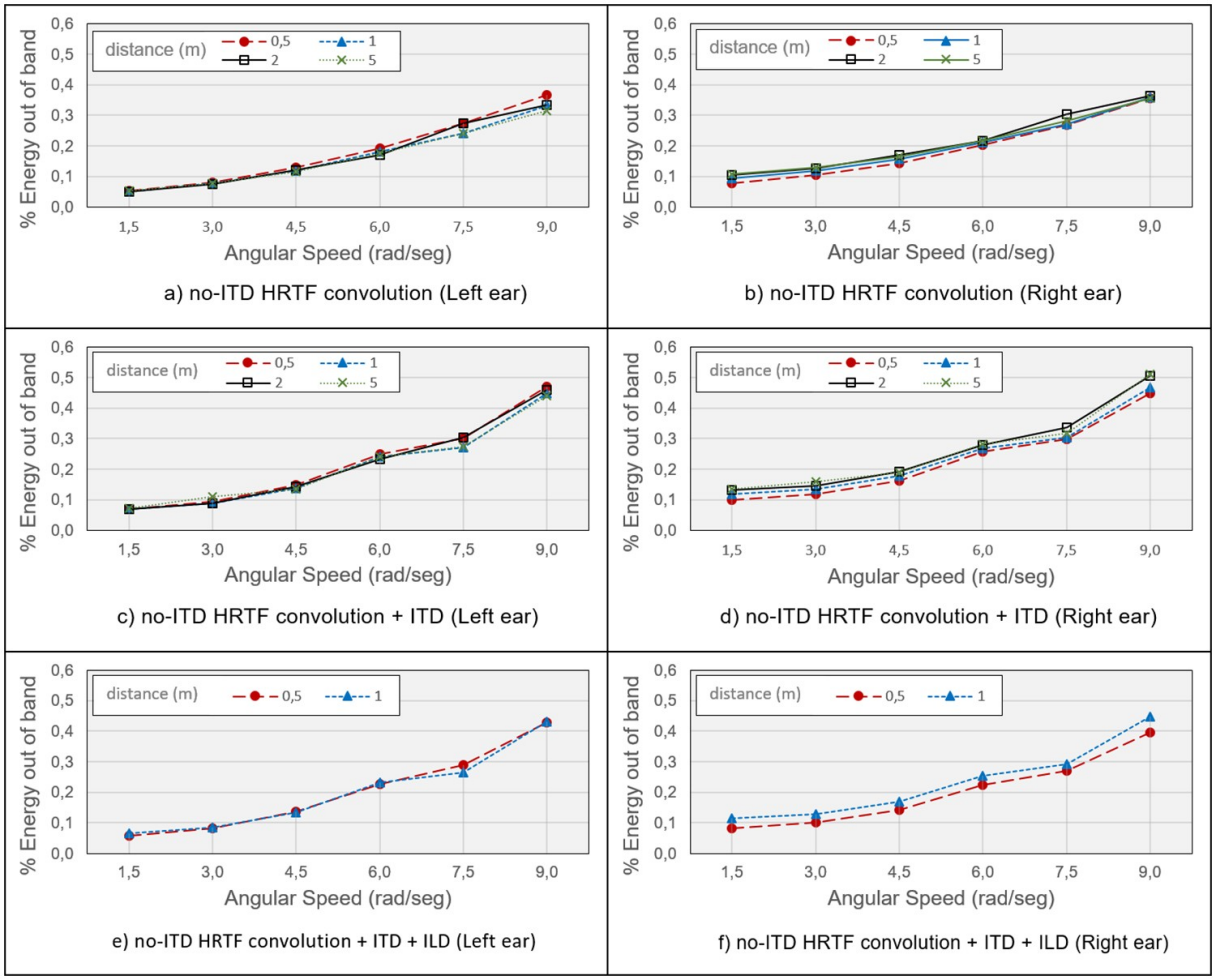
Energy out of band produced by the spatialization process for different configurations. a) and b): only convolution with no-ITD HRTF, c) and d): convolution with no-ITD HRTF and computed ITD, e) and f) convolution with no-ITD HRTF and computed ITD and ILDs. Every configuration for both ears.

**Fig 16:**
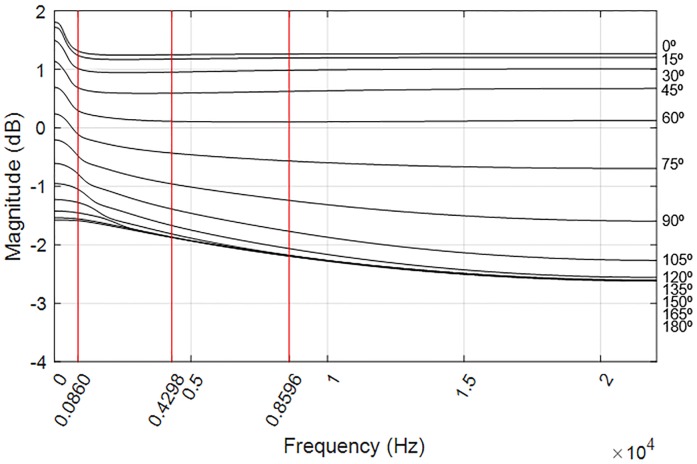
Difference filters implemented for near-field HRIR correction. Vertical lines indicate the frequency of the tones used for the evaluation. The vertical right axis indicates the interaural azimuth of each filter.

EoB was calculated for each combination of distance and speed, giving the results in Fig 15, which shows the EoB for different source distances and angular speeds: Fig 15a and 15b display the EoB, for the left and right ear respectively, when the no-ITD HRTF is used, but without any further ITD processing. It can be noted that increasing speed results in an increased EoB, as expected, but even at high speed (9 rad/s) the overall distortion is relatively small. Fig 15c and 15d refer to the use of no-ITD HRTFs with ITDs added after the interpolation and convolution, simulating a spherical head with 8.75 cm of radius. The estimated distortion shows only a very slight increase if compared with the previous conditions, despite the fact that a delay of up to 30 samples is applied, which dynamically decreases down to 0 and up again on the other ear for every full lap. Finally, Fig 15e and 15f refer to the use of no-ITD HRTFs, synthesised ITDs and near-field ILD correction. Only distances under 2 meters are plotted here, as the near-field compensation is activated only in that distance range. In this case, EoB does not increase when adding the near-field correction; on the contrary, a minor decrease of the overall distortion is noted. This is probably due to the fact that non-linear distortion is higher in the contralateral ear, where near-field correction filters apply higher attenuation, as can be seen in Fig 16. In any case, distortion introduced by the dynamic behaviour of these filters can be considered negligible in the light of Fig 15.

## Discussion and comparison with existing tools

There are many tools available for binaural spatialisation, both commercial and open-source. Some are provided by major actors in the virtual reality industry, such as Google or Oculus (owned by Facebook). There are as many implementation approaches, feature sets and license schemes as there are tools. In this section, we focus our comparison of the characteristics of the 3DTI Toolkit with those of the most representative open-source tools. In addition, we also compare the 3DTI Toolkit with the most popular commercial, closed-source tools. Nevertheless, in some cases it has not been possible to gather detailed information about the various algorithms, as they are not reported in the available documentation.

Tables 2 and 3 give a summarised overview of the various features and characteristics of binaural spatialisation tools, including the 3DTI Toolkit. As mentioned earlier, for closed-source tools it has not always been possible to gather information about which approach/algorithm/technique they use. In some cases, we were able to infer this information from comments in the API code. When this was not possible, we used the abbreviation NR (not reported) for the specific feature. Thus Table 3 is not as reliable as Table 2.

**Table 2:** Comparison of the 3DTI Toolkit with the most popular open-source audio spatialisation tools.

Tool	Direct Path	Real-Time Interp.	HRTF Import	Custom ITD	Near-Field	Reverberation	License	Ref.
3DTI Toolkit	HRTF; SHM	Yes	Yes (SOFA, own format)	Yes	Yes (Ear parallax & ILD correction based on SHM)	CBR (Ambisonic encoded BRIR)	GPL v3	
Resonance audio	AMBI	NA	No	No	Yes (bass boosting)	Ray tracing for arbitrary geometries and SMD	Apache 2.0	[93]
Csound	HRTF; SHM	Yes	No	Yes	No	FDN (shoe-box with configurable number of reflections)	LGPL v2.1	[94]
Slab3d	HRTF	Yes	Yes (own format)	Yes	No	Up to 6 spatialised early reflections in a shoe-box	NOSA v1.3	[95]
OpenAL Soft	HRTF	Yes	Yes (own format)	Yes	Yes (Bass boosting)	FDN (configurable impulse response parameters)	LGPL v2	[96]
Soundscape Renderer	HRTF	No	Yes (WAV)	No	Not for binaural (just NFC-HOA)	CBR using a BRIR file (own format)	GPL v3	[97]
earplug	HRTF	Yes	No	No	No	No	GPL	[98]
hrir	HRTF	Yes	No	No	No	No	GPL	[99]

AMBI: Virtual Ambisonic approaches.

SHM: Spherical Head Model approaches to direct path computation (see text).

NA: Not applicable.

FDN: Feedback Delay Networks

CBR: Convolution Based Reverberation approaches to environment simulation (see text).

NFC-HOA: Near-Field Corrected High Order Ambisonic.

**Table 3:** Comparison of the 3DTI Toolkit with the most popular closed-source audio spatialisation tools.

Tool	Direct Path Approach	Real-Time Interpol.	HRTF Import	Custom ITD	Near-Field	Reverberation	Ref.
3DTI Toolkit (GPLv3)	HRTF; SHM	Yes	Yes (SOFA, own format)	Yes	Yes	CBR (Ambisonic encoded BRIR)	
Oculus	AMBI; HRTF	Yes	No	Yes	Yes	Synthetic approach based on a Shoe-box model (Not many deatils reported)	[100]
MS HRTF	NR	NR	No	NR	NR	Not reported model. Three different sizes of room.	[101]
Rapture 3D	AMBI	NA	Yes (SOFA)	No	NR	Synthetic approach based on a modified Moorer-Schroeder reverberator	[102]
Anaglyph	HRTF	Yes	Yes (SOFA)	Yes	Yes	CBR (Ambisonic encoded BRIR)	[63]
Real Space	NR	NR	No	Yes	NR	Synthetic approach based on model of multiple shoe-boxes (Not many details reported)	[103]
3D Sound Labs	AMBI	NA	No	Yes	NR	Synthetic approach (Few details reported)	[104]
Steam Audio	HRTF	Yes	YES (own format)	No	NR	Ray tracing with arbitrary geometry	[105]
SPAT	HRTF; AMBI	NR	Yes (SOFA)	No	Yes	CBR; FDN; Hybrid approach of both.	[44]
VRWorks Audio	NR	NR	No	No	NR	Ray tracing for arbitrary geometries	[106]

AMBI: Ambisonic approaches

SHM: Spherical Head Model approaches to direct path computation (see text).

NA: Not applicable.

NR: Feature not reported

FDN: Feedback Delay Networks

CBR: Convolution Based Reverberation approaches to environment simulation (see text).

### Spatialisation

There are several approaches for rendering the direct and reflected paths when doing binaural spatialisation. The architecture of the 3DTI Toolkit manages the direct and reverberation components as independent modules, which can be connected (or not) at some point in the processing chain. This unique architecture allows for insertion of effects/processes only in the direct or reverberation components, which would not be possible if using other architectures. An example is the near-field ILDs modification, which is applied only to the direct path signal, and not to the reverberation.

#### Anechoic path

There are three main approaches for rendering the direct path:

- **HRTF**. Spatialisation is performed by convolving the source signal with an HRIR, extracted from a given HRTF, or interpolated among different HRIRs from that set. Different approaches to perform the interpolation are employed. Tools such as Soundscape Renderer select the nearest HRIR without interpolating. In this case the spatial resolution of the HRTF measurement is very relevant.
- **AMBI**. Sound sources are encoded into a set of Ambisonic channels, which are subsequently decoded into a set of virtual loudspeakers. Finally, those virtual loudspeakers are spatialised as static virtual sources by convolving their respective signals with the corresponding HRIR, which can be obtained from an HRTF. Notice that different Ambisonic orders can be used, allowing for a variable level of spatial resolution. Typically, renderers can be configured to use up to 3^rd^ order Ambisonic (16 channels). Using higher order Ambisonic results in higher spatial resolution, at a higher computational cost.
- **Spherical Head Model (SHM)**. Spatialisation is synthetically simulated by applying delays to simulate ITDs, and filters to simulate ILDs. These filters are designed according to mathematical models of sound propagation around a rigid spherical head (e.g. [81]). Typically, this approach uses low order IIR filters which are able to capture the ILDs of a spherical head, and allow to process the signals very fast and at low computational costs, at the expense of lower spatialisation quality.

#### Real-time interpolation

The 3DTI Toolkit can load HRTFs with any HRIR distribution, as explained previously. To minimize discontinuities and artefacts when HRIR data is not available for a specific location (due to the HRTF resolution or limitations in the sampled volume), the HRTF grid can be resampled using an offline process, and HRIRs can be interpolated at runtime. The 3DTI Toolkit provides both mechanisms, allowing full customization of the HRTF resampling step. This specific feature could only be found in Slab3d.

#### Reverberation

Most available tools can simulate reverberation, employing two main approaches:

- **Convolution-based reverberation (CBR)**. Impulse responses of the environment to be simulated are convolved with the audio signal. Usually, these impulse responses are binaurally registered using a dummy head microphone, with the sources placed at different locations. This allows for a certain level of spatialisation of the reverberation sound. The main problem of this approach is the computational cost, due to the fact that these impulse responses can be very long. Therefore, different approaches are used to make the process more efficient.
- **Synthetic reverberation**. The response of the room can be simulated synthetically using several different approaches, which can be classified into two categories:
  - Ray tracing, which is normally used only for early reflections and can handle rooms with arbitrary geometry.
  - Other approaches, mainly based on Feedback Delay Networks (FDN) in the time domain, or Spectral Magnitude Decay (SMD) in the frequency domain. These approaches work for late reverberation as well, and are able to simulate simplified geometries, as a *shoe-box* room.

Reverberation simulation in the 3DTI Toolkit consists in the convolution of an Ambisonic sound field with binaural room impulse responses (BRIR), which makes computational cost independent of the number of sources. This solution is combined with the implementation of UPOLS convolution, allowing to spatialise several sources with large BRIRs at the cost of reducing spatial resolution, which is assumed to be less relevant for reverberation processing as opposed to direct path spatialisation. Although BRIR convolution is a solution adopted in other tools (SoundScape Renderer and Anaglyph), only the 3DTI Toolkit allows reading BRIR data from standard SOFA (AES69-2015) files.

Most of the other tools implement synthetic reverberation using parametric shoe-box models, allowing the user to configure the dimensions and materials of a rectangular room, usually processing separately early reflections and late reverberation tail (Oculus spatialiser, 3D Sound Labs, Slab3d, Dear VR). The solution adopted by Real Space 3D Audio is based also on the shoebox model, but allowing to build more complex room geometries by dividing the geometry into multiple shoe-boxes. Some tools go even further, allowing configuration of arbitrary room geometry through physical models of sound propagation based on scene geometry (Steam Audio, Resonance Audio).

Some tools provide means for simulating occlusions and reflections on obstacles (Resonance Audio, Real Space 3D Audio, Steam Audio), while others delegate this to the application level. The 3DTI Toolkit does not provide specific tools for occlusion simulation, but its modular architecture with full separation between direct path and reverberation allows for a straightforward implementation at application level, as in [107].

#### Alternative spatialisation modes

Given the high computational cost of HRTF convolution, many tools provide some options for increasing performance at the cost of lowering spatialisation quality. The 3DTI Toolkit provides two spatialisation modes. The high quality mode implements HRTF convolution with an efficient UPOLS convolution implementation [70]. The high performance mode uses a Spherical Head Model [81] to adjust a 4^th^ order IIR filter which is designed for different interaural azimuth and distances, and stored in a look-up table which can be loaded as a file. This allows to account for near-field effects, as the spherical head model can be computed also for small distances. In addition, the model can be changed by the user by loading another table, a feature not found in other tools. Other tools provide some options for increasing performance at the cost of much lower spatialisation quality. Typical solutions include: implementing distance culling, as far sources are not rendered (OpenAL Soft, Real Space 3D Audio, Dear VR); projecting all sources into an Ambisonic sound field with configurable order (Resonance Audio, 3D Sound Labs, Oculus spatialiser); and using simple stereo panning as a low quality alternative for secondary sound sources (Rapture 3D). It is also common to save resources in the reverberation process, by reducing the number of early reflections (Real Space 3D Audio, 3D Sound Labs), or precomputing room impulse responses for different points in the scene (Steam Audio). Rapture 3D provides also a simplified HRTF model which simulates frequency-independent ILD and ITD.

#### Listener and sound models

A unique feature of the 3DTI Toolkit is the customization of the listener head directionality pattern (from omnidirectional to different cardioid shapes), feature designed mainly for its integration with the simulation of hearing aids. Regarding the modelling of audio sources, the 3DTI Toolkit models sources as points from which sound emanates omnidirectionally. Although this is a common approach in most tools, some go beyond with the simulation of volumetric sources (Oculus, Resonance Audio) and/or directional sources (Resonance Audio, OpenAL Soft, Microsoft HRTF, SPAT).

#### Distance simulation

Most of the other existing available tools simulate distance through level attenuation due to sound propagation through air, which follows the inverse square law (attenuation of 6 dB with every doubling of the distance). Although this is often the default setting due to its physical correctness, some tools provide customization of the distance attenuation curve (Real Space 3D Audio, OpenAL Soft). The 3DTI Toolkit implements the inverse square law, but allows for customization of the attenuation slope (in dB every double distance), which is a solution found also in a few other tools (Rapture 3D, SoundScape Renderer). Furthermore, the 3DTI Toolkit implements a completely independent management of the attenuation for the direct and the reverberation path, allowing distance-dependent changes in the direct-to-reflected signal ratio.

The effect of air absorption at high frequencies for large distances (e.g. more than 15 metres) has been addressed by some tools, including the 3DTI Toolkit (Oculus spatialiser, Steam Audio, Rapture 3D, Slab3d), while others just provide distance culling to save processing resources for far sources (OpenAL Soft, Real Space 3D Audio, Dear VR).

Regarding simulation of near-field sources, Oculus spatialiser and Resonance Audio model the effect of acoustic diffraction around the head, and SoundScape Renderer provides a resource-expensive experimental solution using High Order Ambisonic (HOA) [108]. As mentioned before, the 3DTI Toolkit uses a set of IIR filters to perform the near-field correction of HRTF together with considering cross-ear parallax, which is the same approach used by SPAT and Anaglyph.

### HTRF customization

Many existing tools do not allow any mechanism for HRTF customization, providing instead one fixed average HRTF (CSound, Resonance Audio, Oculus) or a choice between a few presets (Real Space 3D Audio, Rapture 3D). Other tools provide an SDK with data structures that can be filled by coding from scratch a file reader (Steam Audio). There are also some research on customising the HRTF based on pictures of the ear, like the system announced by 3D Sound Labs [109]. Also Real Space 3D Audio works on HRTF personalization based on anthropometric measurements and machine-learning for automatic selection from existing HRTF databases. These features are very promising, but still in prototype/evaluation stage, and not included in any stable library. Microsoft HRTF uses a fixed standard HRTF (averaged from anthropometric measures) for elevation cues, but allows parameterisation of the listener for azimuth spatialisation.

#### HRTF import

The 3DTI Toolkit API allows customization of the HRTF via a set of methods to load HRTF files from the standard file formats AES69-2015 (also known as SOFA), our own format (.3dti) or directly by loading an array of floats for the impulse responses using the SDK. Other tools which able to load any HRTF in SOFA format are Rapture 3D and Anaglyph. Slab3d, 3D Sound Labs and OpenAL Soft allow loading HRTFs only using their own format. Soundscape Renderer imports multichannel WAV files, with two channels for each direction, but building this set of WAV files is a long and complex process.

A few tools have converted some HRTF databases to their own custom format; Slab3d has translated the LISTEN and CIPIC, SoundScape Renderer has translated FABIAN and KEMAR and Rapture 3D have translated a subset of LISTEN. Using custom file formats implies that the users cannot (or hardly can) do their own translations of HRTFs, and need to rely the designers of each tool for this task.

#### Customizable ITD and near-field correction

Beyond HRTF customization, the 3DTI Toolkit allows customization of listener head radius for ITD processing. In most tools, the delays for ITD are implicit in the HRIR data and cannot be configured separately, except for some notable exceptions (Slab3d, OpenAL Soft, Anaglyph, Oculus).

In addition, the 3DTI Toolkit allows loading near-field filter data for HRTF correction (using custom.3dti-ild files). These filters are based on a rigid spherical head model, and are applied after convolution with the interpolated HRIR. Moreover, a cross ear parallax correction is applied, which is especially relevant for near-field sources. Some other tools, like SPAT or Anaglyph, use this technique but, to the best of our knowledge, no other open-source tool allows this realistic near-field HRTF correction.

## Releases

The 3DTI Toolkit is available on a public GitHub repository (https://github.com/3DTune-In/3dti_AudioToolkit) under a GPLv3 licence. It consists of a C++ library which uses only platform-independent code from the standard template library. The only third-party library integrated in the 3DTI Toolkit is the Takuya Ooura General Purpose FFT [110]. All file read and write operations are provided in an optional separate package (see Additional tools).

The 3DTI Toolkit has been successfully tested in the Windows, MacOS, Linux, Android and iOS operating systems. A complete example project on how to build a simple application which uses the 3DTI Toolkit to spatialise audio is shared (https://github.com/3DTune-In/3dti_AudioToolkit_Examples) under GPLv3 as well.

The 3DTI Toolkit has also been partially embedded in JavaScript through transcompilation (functions such as reverberation and reading of SOFA and.3dti files containing HRTFs are not yet supported). The JavaScript wrapper available as open-source in another GitHub repository (https://github.com/3DTune-In/3dti_AudioToolkit_JavaScript), and has been used to create a web-based version of the 3DTI Toolkit, available at http://online-toolkit.3d-tune-in.eu/.

A 3DTI Toolkit release as Unity3D package has also been created, but is currently being evaluated, and not yet available to the general public. If interested in this release, please contact the authors.

Finally, a set of demonstrator test applications, presented in section Additional tools, have been released which allow users to access the 3DTI Toolkit features through a simple but comprehensive user interface. The test applications can be downloaded from the GitHub repository (https://github.com/3DTune-In/3dti_AudioToolkit/releases) and are available for the Windows, MacOS and Linux operative systems. In addition, an audio samples are available in the public repository where the spatialisation capabilities of the 3DTI Toolkit can be experienced (https://github.com/3DTune-In/3dti_AudioToolkit/tree/master/docs/audio_demo).

## Conclusions

In this paper we presented the 3DTI Toolkit, an open-source C++ library for binaural spatialisation. 3D audio is increasingly being used in VR and gaming applications, and a large amount of research has been conducted in the recent years on this topic. This resulted in several 3D audio rendering tools to be released, with various characteristics and integrating different features. However, not all of them are available as open-source. As Ince and colleagues argue in a recent editorial in Nature [111], the rise of computational science has added a new layer of inaccessibility. This should be overcome by the release of computer programs as open-source, allowing clarity and reproducibility. And this is exactly one of our main aims and one of the reasons we decided to create and release the 3DTI Toolkit.

Among the tools currently available as open-source, the 3DTI Toolkit is the one allowing most configurability, making it a very appropriate instrument for 3D audio research. To the best of our knowledge, it is the only open-source one accepting custom HRTFs in the standard SOFA format for the anechoic path, and custom BRIRs for the simulation of the reverberation.

Furthermore, it is important to emphasize that, having been implemented according to the C++ 14 standard, the 3DTI Toolkit is highly portable. As an example, the demonstrator test application is currently available for Windows, Mac and Linux, and the Toolkit has also been tested for Android and iOS. Moreover, the anechoic process path has been trans-compiled to JavaScript using Emscriptem [112], and an on-line demonstrator is available (http://online-toolkit.3d-tune-in.eu/).

As it has been outlined in the paper, a special effort has been put in removing artefacts related to dynamic scenes, where sources and listener are free to move. This is a particularly important condition for interactive VR applications where the sound designer cannot easily predict scene changes in advance. Furthermore, considering that real-time convolution with HRTFs and BRIRs can become an issue in terms of computational costs, the use of Uniformly Partitioned Convolution is a very unique feature that, to our knowledge, has not yet been implemented in other available open-source tools.

Another feature of the Toolkit, again very relevant for VR applications, is the simulation of near-field effects. The user is completely free to move and approach sound sources in the virtual environment, which is rendered simulating both directional and distance cues to a level of accuracy which cannot be found in other available tools.

Finally, the 3DTI Toolkit implements some unique custom features such as configurable listener/microphone directionality, which allows to simulate the presence of devices such as hearing aids. In this context, it is important to underline that the 3DTI Toolkit also includes simulators of hearing loss and hearing aid [64]. These features are out of the scope of this paper, and will be detailed in further publications.

The 3DTI Toolkit is an alive project, which is being continuously improved and assessed. This is obviously facilitated by its open-source nature, which allows for external contributions and bug reporting. Our plans for future developments include, for example, improving the customization by computing ILD compensation for near-field effects on-line, instead of relying in a pre-computed filter. We are also planning to add multi-listener support, which would allow the use of binaural sound in collaborative virtual environments. Further work on assessing the 3DTI Toolkit performances is also being planned, both in terms of signal processing and perceptual/subjective attributes (e.g. realism, audibility of processing artefacts, etc.), including comparisons with other existing tools.
